# Notch Signaling Mediates the Age-Associated Decrease in Adhesion of Germline Stem Cells to the Niche

**DOI:** 10.1371/journal.pgen.1004888

**Published:** 2014-12-18

**Authors:** Chen-Yuan Tseng, Shih-Han Kao, Chih-Ling Wan, Yueh Cho, Shu-Yun Tung, Hwei-Jan Hsu

**Affiliations:** 1 Institute of Cellular and Organismic Biology, Academia Sinica, Taipei, Taiwan; 2 Graduate Institute of Life Sciences, National Defense Medical Center, Taipei, Taiwan; 3 Genomic Core Facility, Institute of Molecular Biology, Academia Sinica, Taipei, Taiwan; Stanford University Medical Center, United States of America

## Abstract

Stem cells have an innate ability to occupy their stem cell niche, which in turn, is optimized to house stem cells. Organ aging is associated with reduced stem cell occupancy in the niche, but the mechanisms involved are poorly understood. Here, we report that Notch signaling is increased with age in *Drosophila* female germline stem cells (GSCs), and this results in their removal from the niche. Clonal analysis revealed that GSCs with low levels of Notch signaling exhibit increased adhesiveness to the niche, thereby out-competing their neighbors with higher levels of Notch; adhesiveness is altered through regulation of E-cadherin expression. Experimental enhancement of Notch signaling in GSCs hastens their age-dependent loss from the niche, and such loss is at least partially mediated by Sex lethal. However, disruption of Notch signaling in GSCs does not delay GSC loss during aging, and nor does it affect BMP signaling, which promotes self-renewal of GSCs. Finally, we show that in contrast to GSCs, Notch activation in the niche (which maintains niche integrity, and thus mediates GSC retention) is reduced with age, indicating that Notch signaling regulates GSC niche occupancy both intrinsically and extrinsically. Our findings expose a novel role of Notch signaling in controlling GSC-niche adhesion in response to aging, and are also of relevance to metastatic cancer cells, in which Notch signaling suppresses cell adhesion.

## Author Summary

Aging is frequently associated with a decline in the size of stem cell pools, but little is known regarding the molecular mechanisms underlying this process. Here, we report that Notch signaling is increased in GSCs as they age, and this promotes their removal from the niche in an E-cadherin dependent manner. In contrast to GSCs, niche cells exhibit decreased Notch signaling with age; Notch signaling in these cells controls niche integrity, and consequently GSC retention. While Notch signaling in the niche is regulated by insulin signaling, Notch signaling in GSCs is controlled by Sex lethal, an RNA-binding protein. These results imply that Notch signaling is regulated in a cell-type-dependent manner, and coordination between GSCs and their niche facilitates the removal of cells from the niche during the aging process.

## Introduction

Age-associated depletion of stem cell pools has been reported for mammalian satellite stem cells, *Drosophila* male and female GSCs, and *C. elegans* GSCs [1]–[4]; however, the mechanisms underlying such depletion remain unknown. The stem cell niche houses stem cells and maintains their cell identity, by providing physical contact and stemness factors, respectively [5]. In addition to the niche, stem cell-intrinsic factors also regulate stem cell function [6], [7]. These signals are tightly coupled, and regulate stem cells to fit the current needs of the organism. During aging, diminished niche function leads to stem cell loss [1]; on the other hand, it is unknown whether stem cells influence their own attachment to the niche as they age. Moreover, it is also unclear how niche cells coordinate with stem cells in response to aging.

**Figure 1:**
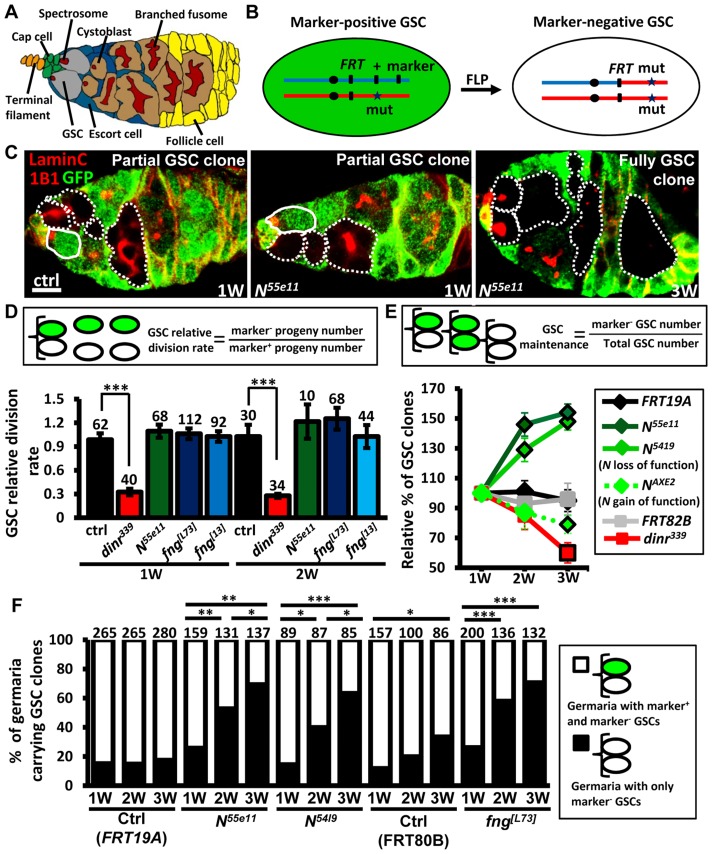
Mutation of *fng* or *N* enhances GSC maintenance without affecting proliferation. (A) *Drosophila* germarium. The GSC niche, which consists of a terminal filament, cap cells, and anterior escort cells, houses two GSCs, which contain membranous organelles (spectrosomes or fusomes). One GSC division gives rise to a cystoblast, which then develops into cyst cells that are interconnected with a branched fusome. The cyst is then surrounded by somatic follicle cells. (B) Mitotic recombination was used to generate GSCs mutant for *N* or *fng*. Females carrying a wild-type (wt) allele linked to a marker gene (*GFP* or *ß-gal*) *in trans* with a mutant (mut) allele were generated. FLP-mediated recombination between *FRT* sites during mitotic division generates a homozygous mutant cell, identifiable by the absence of marker expression. (C) Mosaic germaria with GFP (green, wt cells), 1B1 (red, GSC fusomes) and LamC (red, cap cell nuclear envelopes) labels. Wt GSCs are outlined by solid lines. GFP-negative GSCs and their daughter cells are outlined by dashed lines. One-week (w)-old control (ctrl) and *N^55e11^* mutant germaria contain one wt and one GFP-negative GSC (partial GSC clone) and their progeny. Three-w-old *N^55e11^* mutant germaria contain two GFP-negative GSCs (full GSC clone) and their progeny. Scale bar, 5 µm. (D) GSC relative division rate (ratio of marker-negative to marker-positive GSC progeny) in mosaic germaria. The number of GSCs analyzed is shown above each bar. (E) Relative percentage of GSC clones (as a proportion of total GSCs) at 1, 2, and 3w after clone induction. (F) Relative percentage of germaria carrying partial GSC clones vs. germaria carrying full GSC clones at 1, 2, and 3w after clone induction. The number of germaria analyzed is shown above each bar. *, *P*<0.05; **, *P*<0.01; ***, *P*<0.001. Error bars, mean ± SEM. The genotype of the control germaria in E and F is *hs-flpneoFRT19A/ubiGFPFRT19A* (*FRT19A*). *FRT80B* and *FRT82B* refer to *hs-flp/+; neoFRT80B/armlacZFRT80B* and *hs-flp/+; FRT82Bneo/FRT82BarmlacZ*, respectively.

*Drosophila* is a small organism with a short life span; such properties, combined with the availability of powerful genetic approaches, making this organism eminently suitable for investigations into cellular and organismic responses during aging. In addition, the *Drosophila* ovary houses well-characterized GSCs and their niche (Fig. 1A) [8]. These advantages make the *Drosophila* ovary an excellent model in which to study the communication of stem cells with themselves and the surrounding environment. One *Drosophila* ovary is composed of 16 to 20 ovarioles, which are the basic functional unit of egg production [9]. The anterior-most structure of the ovariole is called the germarium; the tip of the germarium contains the GSC niche, which is composed of terminal filament, cap cells, and anterior escort cells [10], [11]. GSCs make direct contact with cap cells, a major niche component, through E-cadherin-mediated cell-cell adhesion [12]; the GSC fusome, an organelle with a membranous-like structure, is juxtaposed to the interface between cap cell and GSC [13]. GSC division gives rise to a cystoblast, which subsequently undergoes four rounds of incomplete division to form a 16-cell cyst, in which the cells are interconnected with branched fusomes [9]. The 16-cell cyst is then surrounded by a layer of follicle cells, and eventually develops into a mature egg.

The Notch signaling pathway is highly conserved, and plays critical roles in the regulation of stem cells in different systems [14], [15]. In *Drosophila*, it controls the maintenance of niche cap cells [16], [17]; however, whether it plays a role in GSCs is not clear. *Drosophila* has one Notch receptor (encoded by *Notch, N*), which is a single-pass transmembrane protein. Upon binding to one of its ligands, the cleaved intracellular domain of Notch translocates to the nucleus, where it regulates transcription of gene targets. The extracellular domain of Notch is composed of several epidermal growth factor (EGF)-like repeats, which must be highly glycosylated for proper function of the protein [18]. Fringe (encoded by *fng*) is a glycosyltransferase that adds N-acetylglucosamine onto this domain, thereby modulating the binding of Notch to its ligands.

The insulin/insulin-like growth factor (IGF) signaling pathway is also evolutionarily conserved; this pathway controls various stem cell types in response to nutritional input and aging [8], [19]–[22]. We have previously shown that insulin signaling controls Fringe-mediated Notch activation in the GSC niche, and this is required to maintain niche integrity, thereby supporting the retention of GSCs in the niche [23], [24]. In addition, insulin signaling also directly controls GSC proliferation [25], [26], but it is not clear if Notch signaling is involved in this process.

In this article, we demonstrate that insulin and Notch signaling have independent roles in regulating GSCs, in contrast to the earlier observation that insulin signaling regulates Notch activation in niche cells to support their maintenance [23], [24]. While insulin/IGF signaling is required for GSC proliferation and maintenance, Notch signaling underlies the diminished ability of GSCs to occupy the niche during aging. In young ovaries, Notch signaling in GSCs is low, allowing them to persist in the niche; as ovaries age, Notch signaling in GSCs is elevated, resulting in the loss of GSCs from the niche. In contrast to GSCs, Notch signaling in niche cells (which is required for niche integrity) is decreased with age, thus contributing to age-induced GSC loss. We therefore demonstrate that both intrinsic and extrinsic Notch signaling control the niche residency of GSCs during aging.

## Results

### Notch signaling in GSCs is not required for their division or maintenance

Notch signaling has been previously reported to be required for the integrity of the *Drosophila* female GSC niche, and thus contributes to GSC maintenance [16], [17]. We observed that Notch signals are also present, albeit weakly, in GSCs (S1 and S2 Fig.)[24]; however, the function of Notch signaling in GSCs is unknown. To address this question, we used mitotic recombination to generate GSCs with mutations in *N* (indicated by the absence of GFP) (Fig. 1B and C). We first addressed whether Notch signaling is required for GSC division (Fig. 1D). We counted the number of wild-type versus mutant cystoblasts and cysts present in *N* mosaic germaria containing one wild-type and one mutant GSC. Crucially, the relative numbers of wild-type and mutant cystoblasts or cysts were unaffected by early germline death (S3 Fig.). Because each cystoblast or cyst is derived from one GSC division, the ratio of mutant to wild-type progeny is a measure of their relative division [25]. The number of progeny derived from control GSCs labeled with GFP was approximately equal to those without GFP in mock mosaic germaria, resulting in a relative division rate equal to approximately 1.0 at one week and two weeks after clone induction (ACI). Division was unaffected in GSCs homozygous for *N^55ell^*, a null allele [23] (Fig. 1D); these mutant GSCs exhibited decreased levels of Notch signaling as compared to their neighboring control GSCs (S4 Fig.). We next asked if Notch signaling controls GSC maintenance, by examining the number of germaria carrying *N^55ell^* mutant GSCs over time (S1 Table). At three weeks, 89% of *FRT19A* control germaria (n = 737) retained at least one wild-type control GSC generated from the first week, indicating that up to 11% of GSCs undergo turnover naturally, consistent with an earlier report [27]. Most mutant GSCs, however, were retained at 3 weeks ACI in *N^55e11^* mutant mosaic germaria (115±7%, n = 392), indicating that they are resistant to the age-dependent attrition displayed by wild-type GSCs. These findings demonstrate that Notch signaling is not directly required for GSC division; instead, it may be important for long-term GSC maintenance.

### Notch signaling is differentially regulated in GSCs and their niche

Notch activation mediated by Fringe (a glycosyltransferase modulating Notch for its binding to ligands) is required for the maintenance of *Drosophila* GSC niches [28], and is regulated by insulin signaling in response to diet [23], [24]. Insulin signaling is known to directly control GSC division [25]; indeed, *insulin receptor* (*dinr^339^*) mutant GSCs divided three times slower than wild-type GSCs (Fig. 1D). We also found that only 39.8±7% of *dinr^339^* mutant mosaic germaria carried *dinr^339^* mutant GSCs at three weeks ACI (S1 Table), implying a direct role of insulin signaling in GSC maintenance with age. However, *fringe* mutant GSCs exhibited decreased levels of Notch signaling and behaved similarly to *N^55e11^* mutant GSCs (S4 Fig. and S1 Table), indicating that Fringe-mediated Notch signaling and insulin signaling play distinct roles in GSCs, and Notch signaling in GSCs and niche cells is regulated through different processes.

### Low levels of Notch signaling increase GSC competitiveness for niche occupancy

Surprisingly, a 44% net increase in the proportion of *N^55e11^* mutant GSCs was observed at three weeks ACI (Fig. 1E), while no obvious change was observed in the proportion of wild-type control GSCs in *FRT19A* control germaria (Fig. 1E and S1 Table). Because a niche usually houses two to three GSCs, this relative increase may reflect a loss of neighboring normal GSCs, rather than an increase in the number of *N^55e11^* mutant GSCs. Indeed, the proportion of *N^55e11^* mosaic mutant germaria carrying a mixture of GFP-negative and GFP-positive GSCs (i.e., partial GSC clones) decreased from 77% to 30%, while the proportion of germaria in which all GSCs were mutant (i.e., full GSC clones) increased from 23% to 70% by three weeks ACI (Fig. 1F). In *FRT19A* and *FRT80B* mock mosaic germaria, only 2% and 27% of the observed increase, respectively, was due to the natural loss of neighboring marker-positive GSCs. We confirmed these results using GSCs homozygous for *N^5419^* (a genetic null allele [29]), and also observed similar phenomena in *fng* [*^13^*] and *fng^[L73]^* mutant GSCs (Fig. 1E and F, and S1 Table). These results indicate that GSCs with low Notch signaling are more likely to stay in the niche, while those with higher levels of Notch signaling are more likely to be excluded.

To test the above hypothesis, we generated genetic mosaic females carrying GSCs mutant for *N^AXE2^* (as identified by the absence of GFP), a hypermorphic allele [29]; these mutant GSCs exhibited increased levels of Notch signaling as compared to their neighboring control GSCs (S4 Fig.). As expected, *N^AXE2^* mutant GSCs were lost faster than control GSCs (only 75% of *N^AXE2^* mutant GSCs remained by three weeks ACI) (Fig. 1E and S1 Table). Similarly, constitutive activation of Notch signaling by overexpression of the Notch intracellular domain (NICD) in GSCs also accelerated their loss (discussed later). We consistently failed to detect apoptotic germ cells in germaria carrying *N^55e11^* or *fng^[L73]^* mutant GSCs (see S3 Fig.), suggesting that the lost cells undergo differentiation. However, we cannot rule out the possibility that apoptotic cells were lost too rapidly from the germaria to be detected.

### Notch signaling in GSCs suppresses their adhesion to the niche

**Figure 2:**
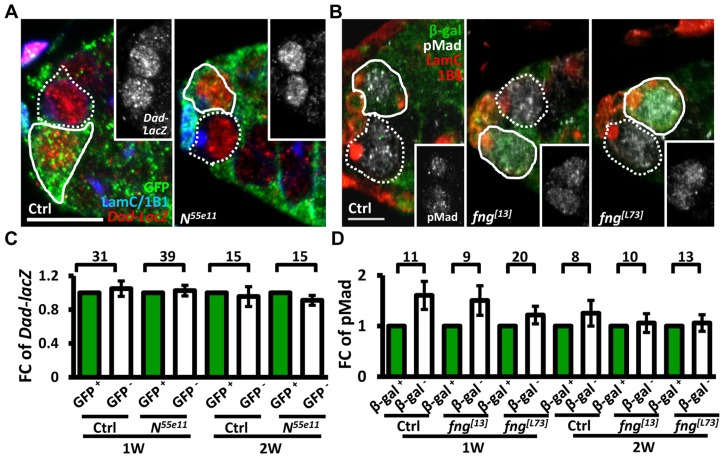
Notch signaling-deficient and control GSCs exhibit comparable levels of BMP signaling. (A and B) One-week (w)-old mosaic germaria. Germaria with GFP (green, wt cells in A), ß-gal (green, wt cells in B), 1B1 (blue, GSC fusomes), LamC (blue, cap cell nuclear envelopes), *Dad-lacZ* (red, A), and phospho (p)-Mad (gray, B) labels. Wt GSCs are outlined by solid lines; control or mutant GSCs (GFP-negative (^−^) in A and ß-gal^−^ in B) are outlined by dashed lines. Scale bar: 10 µm. (C and D) Average fold changes (FC) of *Dad-LacZ* (C) and pMad (D) intensity in GFP^−^ and ß-gal^−^ GSCs, respectively. Values were normalized to those of a wt GSC within the same niche. The number of germaria analyzed is shown above each bracket. The genotype of the control germaria is *hs-flpneoFRT19A/ubiGFPFRT19A* in A and C, and *hs-flp/+*; *neoFRT80B/arm-lacZFRT80B* in B and D.

We next investigated whether enhanced niche occupancy of *N* mutant GSCs is associated with increased Bone Morphogenetic Protein (BMP) signaling, a major pathway for GSC maintenance (Fig. 2). However, the levels of two well-established reporters of BMP signaling [30], phosphorylated (p) Mad (pMad) and *Dad-lacZ*, were unaffected in Notch signaling-defective GSCs, as compared to their neighboring control GSCs.

**Figure 3:**
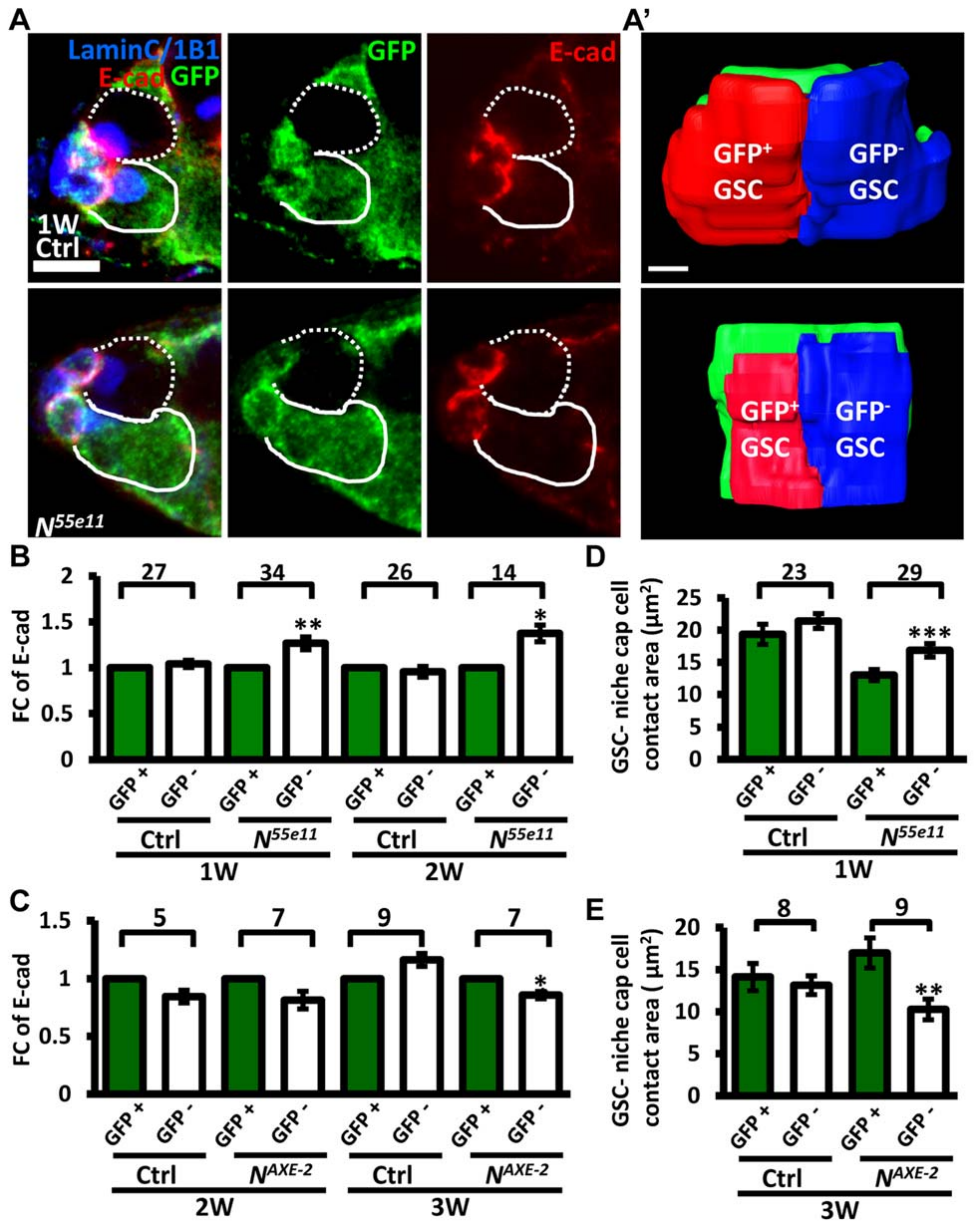
Notch signaling regulates levels of E-cadherin between GSCs and the niche. (A) Mosaic germaria (one-week (w)-old control and *N^55e11^* mutant germaria) with GFP (green, wt cells), E-cadherin (E-cad) (red), 1B1 (blue, GSC fusomes), and LamC (blue, cap cell nuclear envelopes) labels. Wt GSCs are outlined by solid lines; control or mutant GSCs (GFP-negative (^−^)) are outlined by dashed lines. Scale bar, 5 µm. A′, contact area between niche cap cells (green) and wt (red) and GFP^−^ GSCs (blue). Scale bar, 1 µm. (B and C) Average fold changes (FC) of E-cad expression in GFP^−^ GSCs, relative to those of a wt GSC within the same niche of ctrl, *N^55e11^* (B), or *N^AXE-2^* (C) mosaic germaria at 1, 2 or 3w after clone induction. (D and E) Contact area between niche cap cells and GFP^+^ GSCs or GFP^−^ GSCs in ctrl, *N^55e11^* (D), or *N^AXE-2^* (E) mosaic germaria at 1, 2 or 3w after clone induction. The number of mosaic germaria analyzed is shown above each bracket. *, *P*<0.05; **, *P*<0.01; ***, *P*<0.001. Error bar, mean ± SEM. The genotype of the control germaria is *hs-flpneoFRT19A/ubiGFPFRT19A*.

Next, we hypothesized that increased retention of *N* mutant GSCs in the niche may arise from enhanced adhesive ability. E-cadherin is a cell-cell adhesion molecule required for GSC-niche adhesion [12]; we found that its expression was significantly increased in mutant GSC-niche junctions of *N^55e11^* germaria as compared to neighboring normal GSC-niche junctions at one week (98±5 vs. 82±5 arbitrary units, *P*<0.05) and two weeks (91±7 vs. 71±6 arbitrary units, *P*<0.05) ACI (Fig. 3A–B). We also observed that the contact area between *N^55e11^* mutant GSCs and the niche at one week ACI was greater than that between neighboring normal GSCs and the niche (17±1.0 vs. 13±0.4 µm^2^, *P*<0.01) (Fig. 3A′ and D). In contrast, E-cadherin expression and contact area were similar between GFP-positive and -negative GSC-niche junctions in mock mosaic germaria. Germaria containing *fng^[L73]^* or *fng* *^[13]^* mutant GSCs exhibited the same properties as those carrying *N^55e11^* mutant GSCs, but not until two weeks ACI (S5 Fig.); this reflects the relatively moderate effects of these mutations on GSC competitiveness (S1 Table). Conversely, both E-cadherin expression and contact area were decreased at the boundary of *N* gain-of-function mutant (*N^AXE-2^*) GSCs and their niches at 3 weeks ACI (Fig. 3C and E); at this time point, *N^AXE-2^* mutant GSCs were lost more rapidly from the niche than their neighboring control GSCs (see Fig. 1E). These results suggest that low levels of Notch signaling in GSCs increases GSC adhesion to the niche, thereby allowing the cells to out-compete their normal wild-type GSCs.

### Notch signaling controls GSC-niche attachment via E-cadherin

**Figure 4:**
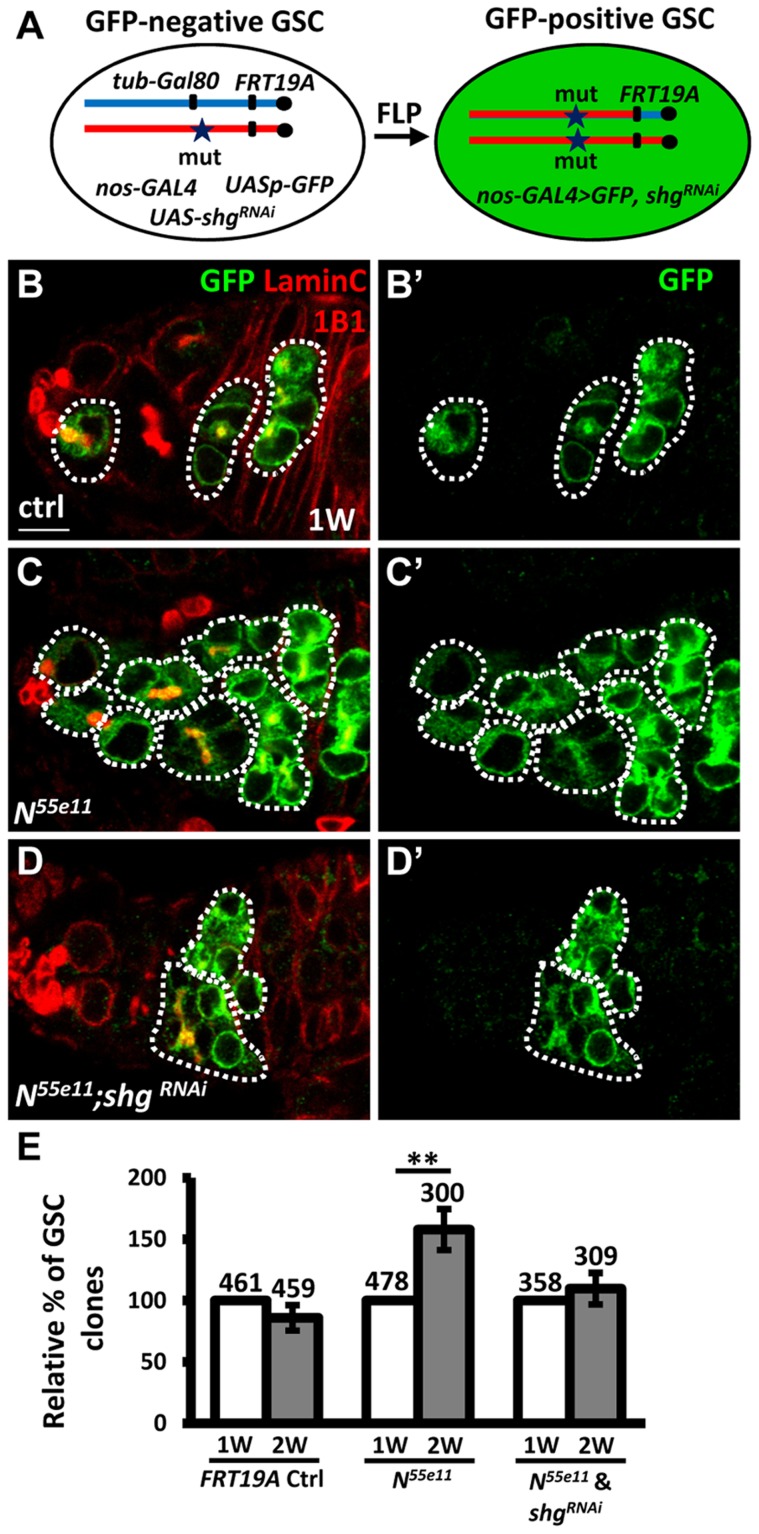
Notch signaling controls GSC-niche adhesion via E-cadherin. (A) Mitotic recombination was used to generate GSCs lacking *N* and *Gal80* (Gal80 suppresses GAL4 expression). Females carried a wild-type (wt) allele linked to a *tub-GAL80* transgene *in trans* with an *N* mutant (mut) allele on one X chromosome, and *non-GAL4*, *UASp-mCD8GFP*, and *UAS-shg^RNAi^* on the third chromosome. FLP-mediated recombination between *FRT* sites during mitotic division generated an *N* homozygous mutant cell lacking GAL80 (identifiable by the presence of GFP expression), which enabled transcription of *GFP* and *shg^RNAi^* via the binding of GAL4 onto UAS elements. (B–D) One-week (w)-old mosaic germaria with GFP (green, mutant cells), 1B1 (red, GSC fusomes), and LamC (red, cap cell nuclear envelopes) labels. GFP-positive (^+^) GSCs and their daughter cells are outlined by dashed lines. Control (ctrl) germarium (B) contains one wt and one GFP^+^ GSC and their progeny, *N^55e11^* mutant germarium (C) contains two GFP^+^ GSCs (full GSC clone) and their progeny, while *shg^RNAi^*-knock down *N^55e11^* mutant germarium (D) contain no GFP^+^ GSCs, but do contain their progeny, indicating that GFP^+^ GSCs have been lost from the niche. B′-D′ show the GFP channel only. Scale bar: 5 µm. (E) Relative percentage of GSC clones (as a proportion of total GSCs) at 1 and 2w after clone induction. The number of mosaic germaria analyzed is shown above each bar. **, *P*<0.01. Error bar, mean ± SEM. The genotype of the control germaria is *hs-flpneoFRT19A/GAL80FRT19A*.

To investigate the requirement of E-cadherin for Notch signaling-mediated niche occupancy of GSCs, we decreased E-cadherin expression in *N^55e11^* mutant GSCs, and then determined the proportion of *N^55e11^* mutant GSCs in mosaic germaria (Fig. 4). To generate *N^55e11^* mutant GSCs expressing RNAi targeting E-cadherin (*shg^RNAi^*), we utilized a combination of FLP-mediated recombination and the binary UAS-GAL4 expression system (Fig. 4A). For these experiments, *Drosophila* females carried a *tub-GAL80* transgene (encoding a suppressor of GAL4 binding to the *UAS* element) located distal to *FRT19A* on one X chromosome, and the *N^55e11^* mutant allele distal to *FRT19A* on the other X chromosome. The product of the *nos-GAL4* transgene was used to drive *UAS-shg^RNAi^* and *UASp-mCD8-GFP* specifically in germ cells [31]. The *tubulin* promoter enables ubiquitous expression of GAL80, and thus GFP and *shg^RNAi^* are suppressed in all cells of the germarium. After recombination, GSCs with two copies of the *N^55e11^* mutant allele would lose *tub-GAL80*, allowing *nos-GAL4* to drive expression of GFP and *shg^RNAi^* (Fig. 4B–D). Consistent with the above findings (see Fig. 1), the proportion of *N^55e11^* mutant GSCs was increased as early as two weeks ACI, as compared with the control (Fig. 4E). Conversely, the proportion of *N^55e11^* mutant GSCs expressing *shg^RNAi^* remained at a similar level to controls (Fig. 4E), indicating that Notch signaling controls GSC competitiveness in an E-cadherin-dependent manner.

### Notch signaling is increased in GSCs during aging

**Figure 5:**
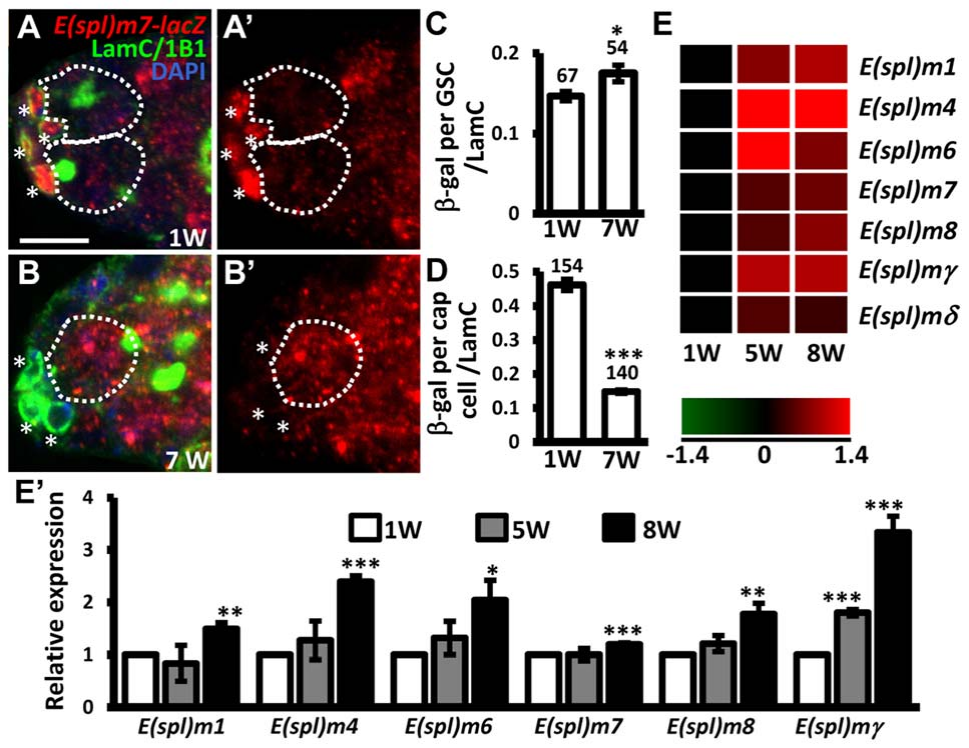
Notch signaling is increased in GSCs with age. (A and B) One-week (w)-old and 7-w-old germaria with ß-gal (red, *E(spl)m7-lacZ*, a *N* reporter), 1B1 (green, GSC fusomes), LamC (green, cap cell nuclear envelopes), and DAPI (blue) labels. A′ and B′ show the *E(spl)m7-lacZ* channel only. GSCs are outlined by dashed circles. Asterisks indicate cap cells. (**C** and **D**) Ratio of ß-gal expression in GSCs (C) and cap cells (D) to average LamC expression in cap cells (internal control) in 1 and 7-w-old germaria. The number of cells analyzed is shown above each bar. **, *P*<0.01; ***, *P*<0.001. Error bar, mean ± SEM. (E) Heat map of *E(spl)* genes that are up-regulated in GSCs with age (*P*<0.05). The colors represent the log 2 values: red - up-regulation; green - down-regulation; black - no change. The gene names are indicated at the right of each row. Each column represents the data from the indicated time point. (E′) Expression levels of the indicated genes in GSCs isolated from 1-week (w), 5w, and 7w-old germaria, as determined using RT-qPCR. Data were normalized to the stable endogenous control gene, *rpL32*. [Data normalized to *rpL19* revealed the same trend at 5w]. Normalized data were subjected to Student's *t*-test. *, *P*<0.05; **, *P*<0.01; ***, *P*<0.001. The genotype of the flies in A–D is +; +; *E(spl)m7-lacZ* *, *P*<0.05; **, *P*<0.001. Error bar, mean ± SEM.

E-cadherin expression is reduced at the junction between GSCs and their niche as germaria age; overexpression of *shg* in GSCs delays age-dependent GSC loss [32], raising the possibility that Notch signaling may be increased in GSCs with age, and mediate suppression of E-cadherin expression. To test this hypothesis, we examined Notch signaling in the GSCs of young and aged germaria using *E(spl)m7-lacZ* (Fig. 5A–D). Expression of *E(spl)m7-lacZ* was approximately 3-fold greater in niche cap cells (n = 154) than in GSCs (n = 67) in one-week-old germaria; *E(spl)m7-lacZ* expression was 68% lower in aged niche cap cells (n = 140) than in young cap cells (n = 154, *P*<0.001), supporting the known role of Notch signaling in niche maintenance [23], which declines with age. However, we observed a 20% increase of *E(spl)m7-lacZ* expression in aged GSCs (n = 54, *P*<0.05) as compared to their younger counterparts. Similar observations were made using a different Notch signaling reporter, *Su(H)Gbe-lacZ* (S6 Fig.) [33]. We also performed microarray analysis of age-dependent changes in transcriptional profiles in GSCs. To this end, we used *bam* mutant females in which the number of GSCs in germaria is increased (Bam is required for GSC differentiation) [34], [35]. We isolated GSCs from 1-, 5-, and 8-week-old *bam* mutant germaria, and performed microarray analyses on three biological replicates (see ‘Materials and Methods' for details). We report that expression of several *Enhancer of split* (*E(spl)*) genes was increased with age in *bam* mutant GSCs, indicating an elevation of Notch signaling (Fig. 5E and E′). These results clearly demonstrate that Notch signaling is up-regulated in GSCs with age.

### Sex lethal suppresses Notch signaling in GSCs

Notch activation requires direct contact between the receptor and its ligands, Delta and Serrate, which are mainly produced in the GSC niche [24]. However, we observed that *bam* mutant GSCs which escaped from the niche still exhibited increased Notch signaling as they aged, suggesting that direct contact between GSCs and the niche is not required for such signaling. Expression levels of Delta (encoded by *Dl*) and Serrate (encoded by *Ser*) were previously reported to be too low to be detected in the germaria [24]; in this study, we did not observe increases in expression of *Dl* or *Ser* in GSCs or their niche (analyzed using two well-characterized reporter lines) (S7 Fig.)[24]. These results suggest that the increase in Notch signaling in GSCs with age is unlikely to be due to increased expression of Notch ligands.

**Figure 6:**
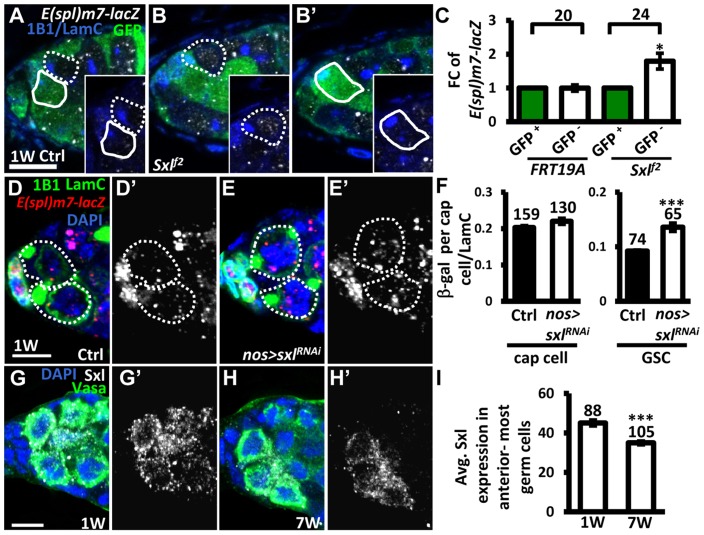
Sxl negatively regulates Notch signaling in GSCs, and its expression is reduced in GSCs with age. (A and B) One-week (w)-old control (ctrl) (A) and *sxl^f2^* mosaic germaria (B) with GFP (green, wild-type (wt) cells), ß-gal (gray, *E(spl)m7-lacZ*, a *N* reporter), 1B1 (blue, GSC fusomes), and LamC (blue, cap cell nuclear envelopes) labels. Wt GSCs are outlined by solid circles, and control or mutant GSCs (GFP-negative (^−^)) are outlined by dashed lines. B and B′ are images from the same germarium, but taken from different focal planes. (C) Average fold changes (FC) of *E(spl)m7-lacZ* expression in GFP^−^ GSCs as compared to those of wt GSCs within the same niche at 1w after clone induction. The number of mosaic germaria analyzed is shown above each bracket. (D–E) One-w-old control (ctrl) (D) and *nos-GAL4-*driven *sxl^RNAi^* germaria (E) with *E(spl)m7-LacZ* (red, *N* reporter), 1B1 (green, fusomes), and LamC (green, cap cell nuclear envelopes) labels. GSCs are outlined by dashed lines. *E(spl)m7-lacZ* signals are shown in gray in D′ and E′. (F) Ratio of average ß-gal signals in cap cells and GSCs to average LamC expression in cap cells (internal control) in 1-w-old ctrl and *sxl*-knock down germaria. (G and H) One-w (G) and 7-w-old germaria (H) with Sxl (gray), Vasa (green, germ cell), and DAPI (blue, DNA) labels. G′ and H′ show the Sxl channel only. (I) Avg. Sxl expression in anterior-most germ cells of 1 and 7-w-old germaria. The number of cells analyzed is shown above each bar in F and I. *, *P*<0.05; **, *P*<0.01. Error bar, mean ± SEM. Scale bars, 5 µm. The genotype of the ctrl is *hs-flpneoFRT19A/ubiGFPFRT19A; E(spl)m7-LacZ/+* in A and C, and *nos-gal4-vp16E(spl)m7-LacZ/+* in D and F.

It is known that Notch signaling is negatively regulated in the developing wing and ovarian epithelial follicle cells by Sex lethal (Sxl; encoded by *sxl*), a female specific RNA-binding protein, via post-transcriptional control of *N* [36]. We therefore investigated whether Sxl also controls Notch signaling in GSCs (Fig. 6). We first generated mosaic germaria carrying GSCs with two copies of *sxl^f2^* (identified by the absence of GFP), a hypomorphic allele [37]. These *sxl^f2^* mutant GSCs exhibited increased Notch signaling (as determined by examining the expression of *E(spl)m7-lacZ*) as compared to their neighboring control GSCs (Fig. 6A–C). Consistent with our finding that elevating Notch signaling in GSCs induces their loss from the niche, *sxl^fs3^* (anothour hypomorphic allele [38]) mutant GSCs were rapidly lost by two weeks ACI (S1 Table). In addition, the use of *sxl^RNAi^* under the control of a germ cell-specific driver (*nos-GAL4*) to knock down *Sxl* expression in GSCs also resulted in a 47% increase of Notch signaling in GSCs (n = 65) as compared to controls (n = 74), *P*<0.01), without affecting Notch signaling in the niche (Fig. 6D–F). These results indicate that Sxl suppresses Notch signaling in GSCs. Of note, we found that ∼20% of *sxl^RNAi^* -knock down and *sxl^f2^* mosaic germaria, and ∼8% of *sxl^fs3^* mutant germaria contained cystoblasts with defects in differentiation (S8 Fig.); this finding is consistent with the known role of Sxl in the control of GSC differentiation [39]. Further, we found that expression of Sxl in the anterior-most germ cells (including GSCs) of germaria decreased with age (1-week-old: 45±1 arbitrary units, n = 88 vs. 7-week-old; 34±1 arbitrary units, n = 103, *P*<0.01) (Fig. 6G–I). Western blot analysis was used to show that Sxl expression in old ovaries is reduced as compared to that in young ovaries (S9A Fig.). Similar observations were also made in isolated GSCs from young and aged *bam* mutant females (S9B Fig.). Our results suggest that the induction of Notch signaling with age in GSCs is at least partly mediated by Sxl.

### Notch signaling in GSCs promotes their removal from the niche

**Figure 7:**
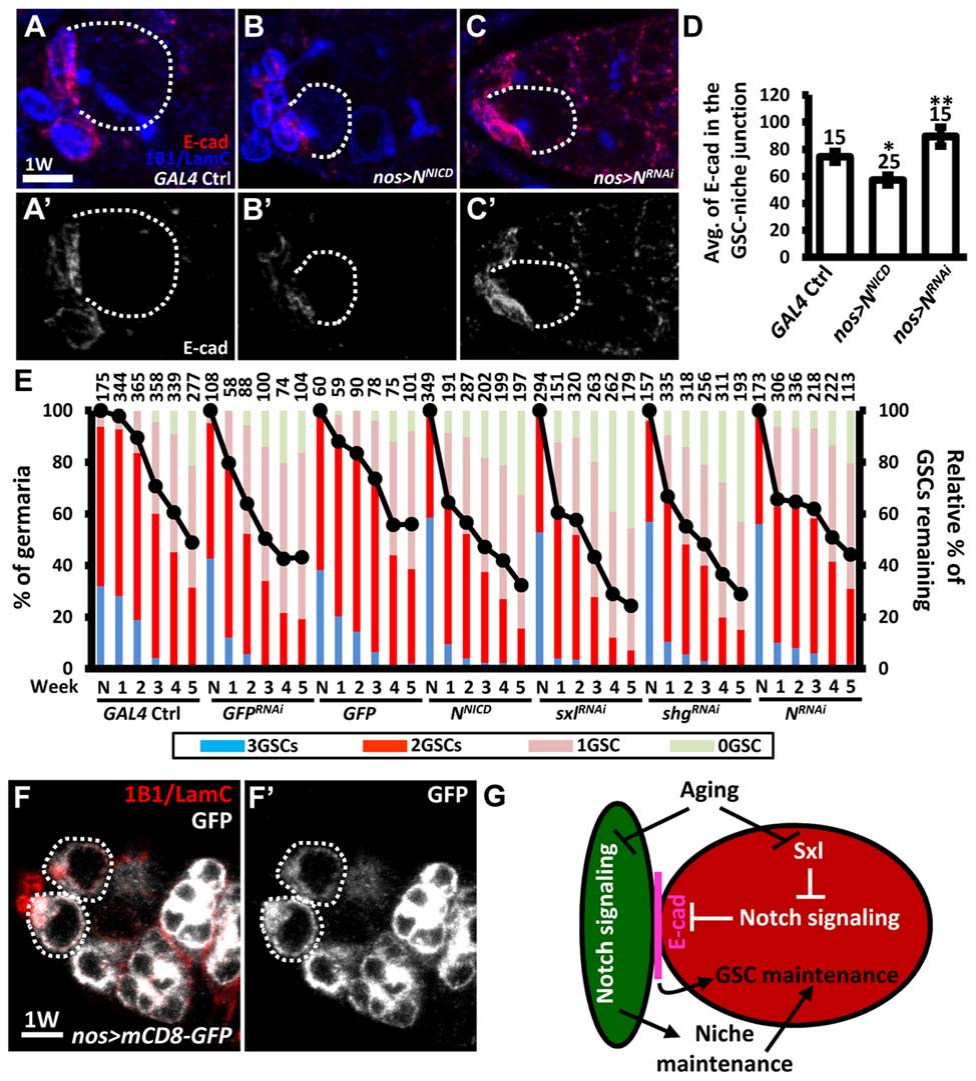
Forced Notch signaling in GSCs accelerates age-dependent GSC loss. (A–C) Three-dimensional reconstructions of images from one-w-old control (A), *nos-GAL4-*driven *N^RNAi^* (B), and *nos-GAL4-*driven *N^NICD^* (C) germaria with E-cadherin (E-cad, red), 1B1 (blue, fusomes), and LamC (blue, cap cell nuclear envelopes) labels. A′-C′ show the E-cadherin channel only. (D) Average (avg.) E-cadherin expression at the GSC-niche junction. The number of GSCs analyzed is shown above each bar. *, *P*<0.05; **, *P*<0.01. Error bar, mean ± SEM. (E) Relative percentage of GSCs remaining in the germaria of the indicated genotypes at different ages. Flies carrying GFP or *GFP^RNAi^* driven by *nos-GAL4* were used as controls. *N^NICD^* was used to increase Notch signaling; RNAi lines were used to suppress the expression of *Sxl*, *shg*, or *N*. The *GAL4* control (ctrl) was obtained by crossing the *GAL4* line with the *w^1118^* strain. Line graphs show the maintenance rates of GSCs with age (N, just eclosed; weeks, shown on the x axis). GSC maintenance rates for each genotype were determined by normalizing the average numbers of GSCs at each time point to that in newly eclosed flies. The maintenance rates of both *N^NICD^*-overexpressing and *Sxl^RNAi^*-knock down GSCs were significantly different from those of all the controls (*P*<0.05), as determined by Wilcoxon Signed-Rank test. Bar graphs indicate the distribution of germaria with 0, 1, 2, or 3 or more GSCs at the indicated weeks. The number of germaria analyzed is shown above each bar. (F) One-w-old *nos-GAL4-*driven *UASp-mCD8-GFP* germaria with GFP (gray), 1B1 (red, fusomes), and LamC (red, cap cell nuclear envelopes) labels. F′ shows the GFP channel only. Scale bars in A and F are 5 µm. (G) Notch signaling mediates coordination between GSCs and their niche in response to aging. Aging reduces Notch signaling activity in cap cells, which disrupts niche integrity, and thus affects GSC maintenance; on the other hand, aging up-regulates Notch signaling at the GSC-niche junction (at least in part through Sxl), thereby promoting GSC loss via the suppression of E-cadherin expression. Dark green, cap cell; red, GSC.

We further hypothesized that the elevation of Notch signaling in older GSCs may help to promote GSC loss from the niche. To test this hypothesis, we used an *N* RNAi line or a constitutively-active form of *N* (*N^NICD^*) driven by *nos-GAL4* to alter Notch signaling in the GSCs in the adult, and then examined the number of GSCs at different ages (Fig. 7). Overexpression of *N^NICD^* in GSCs decreased E-cadherin expression at GSC-niche junctions and accelerated aging-dependent GSC loss (Fig. 7A, B, D, and E). We consistently observed that elevation of Notch signaling or knock down of E-cadherin in GSCs using *sxl^RNAi^* or *shg^RNAi^*, respectively, also promoted GSC loss. However, although inhibition of Notch expression increased E-cadherin expression at the GSC-niche junction (Fig. 7A, C, and D), this was not sufficient to suppress age-dependent GSC loss (Fig. 7E), indicating that multiple factors are required for this process.

Although our clonal analysis revealed that Notch signaling is not required for the maintenance of GSCs in the niche (see Fig. 1), GSCs were rapidly lost (40% decrease) from one-week-old females subjected to *N^RNAi^*-knock down; however, by two- to three-weeks, maintenance in knock-down females was better than that of wild-type females, with levels eventually becoming comparable to that in controls. We suspect that varying expression levels of GAL4 among GSCs in the niche (25 germaria were analyzed) created a competitive environment (Fig. 7E), resulting in GSC loss. However, after the first week, Notch expression in *N^RNAi^*-knock-down GSCs in the niche had dropped to the lowest recorded level, resulting in the loss of competitive behavior, together with an increase in E-cadherin expression at GSC-niche junctions; consequently, these GSCs exhibited enhanced retention. Above three weeks in age, other factors (like a reduction in niche-derived stemness signals) may function in parallel with Notch signaling to control GSC aging. We also confirmed our results using a second germline driver, *maternal-tubulin-GAL4* (*mat -GAL4*). Unlike *nos-GAL4*, *mat-GAL4* is expressed evenly in GSCs within the germaria (25 germaria were examined) (S10A Fig.). Although flies carrying a single copy of *mat-GAL4* did not survive longer than 4 weeks after eclosion at the non-permissive temperature (29°C), we were still able to observe that forced Notch signaling accelerated GSC loss with age, and disruption of *N* slowed GSC loss with age (S10B Fig.). Collectively, our findings reveal that Notch signaling promotes GSC aging, and hence such signaling needs to be maintained at a relatively low level in young GSCs for their anchorage to the niche.

## Discussion

Here, we have described a novel role for Notch signaling in the regulation of stem cell-niche contact during aging, in parallel with factors that control stem cell self-renewal (Fig. 7G). In young germaria, Notch signaling is low in GSCs, but high in the niche, thereby ensuring that GSCs are maintained in the niche (E-cadherin levels are high). However, Notch signaling in older germaria is elevated in GSCs but decreased in the niche, thereby reducing GSC-niche adhesion (E-cadherin levels are low). Our results indicate that Notch signaling is differentially regulated in GSCs (via negative regulation by Sxl) and their niche (via insulin signaling, which is decreased with age [23]), and mediates coordination between stem cells and niche for stem cell maintenance in response to aging. These results have broad significance for stem cell therapy, particularly when considered with the well-known effects of aging on reproduction [40] and the high degree of conservation of Notch signaling function [14].

### Enhancement of Notch signaling may be a common feature in certain types of stem cells during aging

Notch signaling plays critical roles in stem cell maintenance, differentiation, and aging [15]; however, the mechanisms by which Notch signaling controls stem cell aging remain unclear. It has been shown that Notch signaling controls myogenesis and neurogenesis via the control of skeleton muscle stem cells and neural progenitors; however, a reduction in Notch signaling impairs these stem cell functions during aging [41], [42]. Unlike muscle stem cells and neural progenitors, we found that Notch signaling is up-regulated in GSCs with age, and this suppresses GSC adhesion to the niche. Increased Notch signaling activity is also observed in mesenchymal stem cells (MSCs) of patients with Hutchinson-Gilford Progeria Syndrome (HGPS), a premature-aging disease; this increase is caused by abnormal activation of Lamin A, which induces nuclear defects and DNA damage [43], [44]. Increased Notch activation in MSCs forces their differentiation toward osteoblasts, but prevents their differentiation into adipocytes. It is possible that GSCs and MSCs share similar gene expression profiles, which are required for activation of Notch signaling in response to aging; this is supported by the detection of germinal transcripts in MSCs [45].

### Aging may induce stem cell competition for niche residency via up-regulation of Notch signaling

The number of stem cells gradually decline during aging [32], suggesting that the remaining GSCs in the niche are more competitive than those that are lost. It has been proposed that under normal conditions, cycling tissue stem cells behave as an equipotent population, in which the balance between differentiation and proliferation is achieved by frequent and stochastic stem cell loss and replacement (a process termed neutral competition), thereby maintaining tissue homeostasis [46], [47]. However, this balance is disrupted during aging, and, assuming that stem cell loss rate is higher than the speed of replacement, this results in stem cell loss. Here, we argue that aging induces the differences among stem cells in the niche which affect niche residency. For example, aged GSCs in the niche accumulate different levels of DNA damage [48], which may result from the accumulation of excessive reactive oxygen species (ROS); this may ultimately lead to GSC loss [32]. In addition, we also observed that GSCs in the niche exhibited various levels of enhanced Notch signaling (S11 Fig.); this suggests that GSCs with higher levels of Notch signaling activity compared to their counterparts in the niche will be lost earlier, because of decreased GSC-niche attachment. Intriguingly, ROS have the ability to activate Notch signaling via the activation of metalloprotease ADAM17, which triggers the release of NICD [49]. Taken together, Notch signaling might serve as a quality control to removal “poor” GSCs from the niche.

### Sxl expression promotes female GSC maintenance by negatively regulating the Notch signaling pathway

Notch signaling is used for cell-fate determination and appropriate stem cell function in several different contexts, while the manner in which it is regulated is different between the two sexes. This arises because Sxl, a female-specific RNA-binding protein, negatively regulates Notch signaling during larval and follicular epithelium development, by binding to *N* mRNA to control Notch translation [36]. In this study, we show that Sxl maintains low levels of Notch signaling in young female GSCs, and elimination of Sxl in GSCs elevates Notch signaling, triggering early GSC loss. In addition, the pattern of Sxl expression and Notch signaling activity in the germ line is reciprocal (see Fig. 6G and S1A Fig.); Sxl is strongly expressed in GSCs and cytoblasts, but weakly in differentiating germ cell cysts, while Notch signaling activity is low in GSCs and cystoblasts, but high in germ cell cysts. These results suggest that Sxl may regulate Notch signaling in female GSCs through a post-transcriptional process present in other cell types. Notably, Notch signaling activities were similar between male GSCs and their progeny (S12 Fig.); this raises the question, ‘how does Sxl modulation of Notch signaling shape the differences between male and female GSCs?’ In addition, it is not clear how Sxl expression is decreased in cells that require higher Notch signaling, such as aged GSCs. It has been shown that transcription of *sxl* is directly regulated by JAK/STAT signaling during embryogenesis [50]. However, unlike Sxl, JAK/STAT signaling is not required for female GSC maintenance [2], [3]. In a study by Vied et al. [4], Hh signaling was observed to suppress the function of Sxl in the female germline; however, Hh signaling activity is decreased during aging [51]. These results imply that Sxl expression in GSCs is controlled by neither JAK/STAT nor Hh signaling.

Although Sxl is a female-specific protein, *sxl* transcripts with the third exon are present in males; these transcripts encode a non-functional Sxl protein with an early stop codon [52]. During development of females, *sxl* is maintained in the on-state via an auto-regulatory feedback loop [53]. In this loop, female Sxl proteins promote their own expression by directing the splicing machinery to skip the third exon of transcripts derived from the constitutive *sxl* late promoter, which is active in both males and females. Aging is frequently associated with down-regulation of pre-mRNA processing factors that are required for alternative splicing [54], raising the possibility that the decrease in Sxl expression in GSCs with age is due to reduced efficiency of splicing. Nevertheless, further investigation is required to pinpoint the mechanism behind this phenomenon.

### The opposing changes in Notch signaling in cap cells and GSCs with age is probably due to cell-type-specific regulation

We observed that Notch signaling in the niche and GSCs was affected by age in different ways; during aging, Notch signaling was decreased in the niche, but increased in GSCs. A subset of niche cap cells make direct contact with GSCs [24], raising the possibility that Notch signaling in niche cells may affect Notch signaling in GSCs, and *vice versa*. However, disruption of Notch signaling in GSCs did not affect Notch signaling in niche cap cells (see S2 Fig.), and knock down of *N* or *Dl* in niche cap cells did not affect Notch signaling in GSCs (S13 Fig.). In addition, our mosaic clonal analysis revealed that within *N^loss of function^* heterozygous mutant niches, *N^loss of function^* homozygous mutant GSCs (lacking GFP) exhibit higher levels of Notch signaling as compared to their neighboring control GSCs (most of which will be heterozygous for *N^loss of function^* alleles, as the recombination efficiency between two *in trans FRT19* loci is less than 30%), indicating that the increase of Notch signaling in GSCs occurs in a cell-autonomous manner. Similar conclusions were reached through GSC clonal analysis using the *N^gain of function^* allele. Further, induction of Notch signaling in GSCs enhances GSC loss, and based on our microarray analysis of *bam* mutant GSCs distal from the niche; we conclude that the regulation of E-cadherin by Notch signaling in GSCs is performed in a cell-autonomous manner. These results also suggest that the mechanisms that regulate Notch signaling in niche cap cells and GSCs are independent of one another. Indeed, while Notch signaling in GSCs is controlled by Sxl, knock down of *sxl* expression in niche cap cells did not affect Notch activation (S13 Fig.). Furthermore, we previously demonstrated that Fringe (which adds sugar to Notch) is required for Notch activation in niche cap cells [55]), while excessive *fng* (induced by Foxo under insulin insufficiency) suppresses Notch activation. Here, we observed that *N* and *fng* mutant GSCs exhibit similar behavior; however, Notch signaling is not decreased in insulin signaling-defective GSCs (S14 Fig.), suggesting that Notch signaling is differentially regulated between GSCs and their niche.

## Materials and Methods

### Fly stocks and culture

*Drosophila* stocks were maintained at 22–25°C on standard medium, unless otherwise indicated. *yw* and *w^1118^* strains were used as wild-type controls. The null *inr^339^*, *bam^1^*, *bam^Δ86^*, *fng* [*^13^*], *fng^[L73]^*, *N^5419^*, *N^55e11^*, hypomorphic *sxl^f2^* and *sxl^fs3^*, and hypermorphic *N^AXE2^* alleles have been described previously [23], [29], [37], [38], [56]–[59]. *UAS-RNAi* lines against *N* (VDRC 27228), *fng* (VDRC 51799), *shg* (VDRC10392), and *sxl* (10853R-3) were obtained from the Vienna Drosophila RNAi center or Fly Stocks of National Institute of Genetics. The efficiencies of *N ^RNAi^*, *fng ^RNAi^*, and *shg^RNAi^* have been previously reported [24], [60], and the efficiencies of the *shg^RNAi^* and *sxl^RNAi^* lines were examined in the ovary (S15 Fig.). The *UASt* vector (which contains the *hsp70* promoter) is suitable for expressing *RNAi* constructs in the female germline line. Although the SV40 sequence located at the 3′UTR region of the *UASt* vector does not stabilize transcripts for nuclear export and protein synthesis, it does not influence transcription and RNA targeting of *RNAi* in the nuclei of germ cells. *Dad-lacZ* was used to monitor BMP signaling [30], and *E(spl)m7-lacZ*, *E(spl)mβ-CD2*, *Notch responsive element (NRE)-pGreenRabbit*, *NRE-pRedRabbit*, *NRE-pBlueRabbit*, and *NRE-pVenusRabbit* reporters were used to monitor Notch signaling [24], [61]. *E(spl)mβ-CD2* and NRE reporters were not detectable in GSCs. The *nanos (nos)-Gal4-VP16* and *meta-GAL4* lines have been previously described [31], [62]. Flies expressing RNAi or other transgenes driven by *nos-GAL4* also carried *tub-GAL80^ts^* to control GAL4 expression [33]; these flies were cultured at 18°C during development, and then switched to 29°C to allow GAL4 expression. *Vasa-GFP* was used to identify germ cells [63]. Other genetic elements are described in Flybase (http://flybase.bio.indiana.edu).

### Transgenic line generation

A fragment of the *Notch* coding region lacking the extracellular domain was removed from the *PIZ-NΔECN* construct (kindly provided by B. DeDecker, University of Colorado Boulder) using the *Not*I and *Kpn*I sites; the fragment was subcloned into the *UASpI vector* (modified from *pUASp*, T. Murphy, NCBI) to create *pUASp-NΔECN* (referred to as *N^NICD^* in this study). To construct *UASp-mCD8-GFP*, the *mCD8-GFP* fragment was amplified from the *UASt-mCD8-GFP* construct using a pair of primers carrying *FseI* and *AscI* sites (sequences are available upon request). The fragment was subcloned into the *UASpI* vector (modified by L. Lee, University of Vanderbilt). Transgenic lines were generated as described previously [64].

### Genetic mosaic analysis

Genetic mosaics were generated by Flipase (FLP)/FLP recognition target (*FRT*)-mediated mitotic recombination [65]. For conventional mosaic analysis, females of genotype *neoFRT19AFLP122/ubiGFPFRT19A*, N*FRT19AFLP122/ubiGFPFRT19AFLP122, *tubGAL80FRT19AFLP122/neoFRT19A*; *hsflp/+; neoFRT80B/arm-lacZFRT80B*, *hsflp/+*; fng*FRT80B/arm-lacZFRT80B, *hsflp/+*; *FRT82Bneo/FRT82Barm-lacZ*, or *hsflp/+*; *FRT82Bdinr^339^/FRT82Barm-lacZ* were generated from standard crosses (N* represents *N^55e11^*, *N^5419^*, or *N^AXE2^*; fng* represents *fng* [*^13^*] or *fng^[L73]^*). For MARCM (Mosaic Analysis with a Repressible Cell Marker) [66], females of genotype tubp-GAL80FRT19AFLP122/N^*^FRT19A; *nos-GAL4vp16 UASp-mCD8-GFP/+*, tubp-GAL80FRT19AFLP122/N^*^FRT19A; *nos-GAL4vp16>UASp-mCD8-GFP&UAS-shg^RNAi^* were generated. To generate GSC clones, two-day-old females were subjected to heat shock for 1 hour at 37°C, twice a day for three days. After heat shock, females raised at 25°C were transferred to fresh food daily until dissection. *N* loss-of-function mosaic mutant females were cultured at 18°C to avoid lethality. Homozygous mutant cells were identified by the absence of ß-gal or GFP in conventional mosaic analyses, but recognized by the presence of GFP in MARCM.

### Immunostaining and fluorescence microscopy

Ovaries or testes were dissected, fixed, and immunostained as described previously [26]. The following primary antibodies were used: mouse anti-Hts (1B1) (Developmental Studies Hybridoma Bank, DSHB, 1∶50), mouse anti-Lamin (Lam) C (LC28.26) (DSHB, 1∶50), Rat anti-E-cadherin (ECAD-2) (DHSB, 1∶3), mouse anti-Sxl (M180) (DSHB, 1∶350), rabbit anti-pMad (Smad3, #1880) (Epitomics, 1∶200), mouse anti-β-gal (Promega, 1∶500), and rabbit anti-GFP (Torrey Pines, 1∶1,000). Mouse anti-NICD and NECD (DHSB) failed to generate specific signals in GSCs. AlexaFluor 488-, 568- or 633-conjugated goat species-specific secondary antibodies (Molecular Probes, 1∶1000) were subsequently used. ApopTag Fluorescein Direct In Situ Apoptosis Detection Kit (Roche) was used as described [26]; the positive controls for this assay were two-day-old *yw* females starved on a diet of sugar and water for two days. Samples were stained with 0.5 µg/ml DAPI (Sigma), mounted in 80% glycerol containing 20.0 µg/mL N-propyl gallate (Sigma), and analyzed using a Zeiss LSM 700 confocal microscope.

GSCs were identified by the anterior position of their fusome (labeled by 1B1 staining), which is juxtaposed to cap cells (cap cell nuclear envelopes were labeled by LamC staining) [24]. Germaria analyzed for (i) GSC division, (ii) expression of *E(spl)m7-lacZ*, *Dad-lacZ*, E-cadherin, and pMad, or (iii) GSC-niche contact area contained only one wild-type GSC and one marker-negative GSC. To measure GSC relative division rates, the number of GFP-positive progeny (cystoblasts and cysts) was divided by the number of GFP-negative progeny in a given germaria. Due to each cystoblast carries a fusome undergoes four more rounds of division to form two, four, 8, and 16-cell cysts; the cells in each cyst remain interconnected by a branched fusome. Therefore, the numbers of fusomes represent the numbers of GFP-negative progeny derived from the GFP-negative GSC, and likewise for the fusomes carried by GFP-positive progeny.

For quantification of fluorescence signals, all clearly-stained germaria were subjected to analysis. For measuring *E(spl)m7-lacZ*, *Dad-lacZ*, pMad, Sxl and LamC expression, Image J was used to measure the average fluorescence intensity (arbitrary units) in confocal Z-sections at the largest GSC cytoplasmic or nuclear diameter. For quantification of E-cadherin and niche-GSC contact area, five to six optical sections (0.6 µm) were taken along the Z-axis of the E-cadherin-expressing interface between cap cell and GSC. The average intensity of E-cadherin signals at the region of contact between a GSC and cap cells was measured using Image J. For niche-GSC contact area, Avizo software (Visualization Science Group) was used to reconstruct and calculate the surface area volume along the Z-axis of the fusome. Statistical analysis was performed using Student's *t*-test.

### Western blot analysis

Twenty pairs of anterior transparent ovaries parts were dissected from one- and seven-week-old flies, or ∼2×10^5^ GSCs were isolated from one-and five-week-old *bam* mutant females flies; cells/tissues were then lysed in RIPA buffer (20 mM Tris-HCl pH 7.5, 150 mM NaCl, 1 mM EGTA, 1% NP-40) supplemented with 2X Complete Proteinase Inhibitor Cocktail, EDTA-free (Roche) on ice for 1 hour. Lysates (60 µg aliquots) were boiled in sample buffer (50 mM Tris-Cl, pH 6.8, 5% ß-mercaptoethanol, 2% SDS, 0.1% bromophenol blue, and 10% glycerol) for 10 minutes, separated by 10% SDS-PAGE, blotted onto a PVDF membrane, and then blocked with 1X Tris-buffered saline containing 0.1% Triton X-100 (TBST, pH 7.5) and 0.5% *bovine serum albumin* (BSA) for 1 hour at room temperature. The blots were incubated with anti-Sxl (M180) (DSHB, 1∶350) and anti-α-tubulin (Sigma T9026, 1: 5000) antibodies at 4°C overnight with shaking. After three 10 minute washes with 1X PBST, the blots were incubated with anti-mouse IgG-HRP secondary antibody (Millipore; 1: 5000) for 1 hour at room temperature, and then washed three times with 1X TBST. Signals were detected and measured using the ECL system (Perkin Elmer), and compared to a molecular weight standard (Thermo).

### Isolation of GSCs by FACS

Several hundred *bam^1^vasa-GFP/bam^Δ86^vasa-GFP* ovaries were dissected in Grace's insect medium (GIBCO) with 10% FBS, and were subsequently incubated with 0.45% Trypsin (Invitrogen) and 2.5 mg/ml collagenase (Invitrogen) for 20 minutes at 25°C with vigorous shaking. Cell suspensions were filtered twice through a 40 µm nylon mesh. Cells were collected by centrifugation at 1000×g for 7 minutes, re-suspended in 1 ml of Grace's insect medium with 10% FBS and 1 mg/ml of propidium iodide, and then immediately sorted by fluorescence-activated cell sorting (FACS) with the Becton Deckinson FACSCalibur using CELLQUEST software. Living GSCs expressing Vasa-GFP were sorted by gating GFP-positive and red-negative cells with the exclusion model. Sorted cells were collected and kept in Trizol reagent (Invitrogen) at −80°C until RNA extraction.

### Microarray and quantitative (q)-PCR

RNA was extracted from 7×10^5^ isolated *bam* mutant GSCs, and 0.2 µg of total RNA was amplified using a low Input Quick-Amp Labeling kit (Agilent Technologies, USA) and labeled with Cy3 (CyDye, Agilent Technologies, USA) during *in vitro* transcription. Cy3-labeled cRNA (1.65 µg) was fragmented to an average size of about 50–100 nucleotides by incubation with fragmentation buffer at 60°C for 30 minutes. Fragmented labeled cRNA was then pooled and hybridized to Agilent Fly custom 4×44 K Microarrays (Agilent Technologies, USA) at 65°C for 17 h. After being washed and dried with a nitrogen gun, microarrays were scanned with an Agilent microarray scanner (Agilent Technologies, USA) at 535 nm for Cy3. Scanned images were analyzed using Feature extraction 10.5.1.1 software (Agilent Technologies, USA); image analysis and normalization software were used to quantify signals and background intensity for each feature. Selected candidates were validated by q-PCR. In brief, total RNA extracted from isolated GSCs was reverse transcribed using the Transcriptor First Strand cDNA Synthesis Kit (Roche). Steady-state mRNA levels were determined using LightCycler 480 Probes Master combined with a Universal Probe library (Roche); each gene was analyzed using the primer pairs and probes listed below:

*E(spl)m1*: probe#87, 5′-CGAAAGGAATAGCGTGCAG-3′ and 5′-AACTTCTCGTGCAGATTCTCG-3′;

*E(spl)m4*: probe#14, 5′-CTCTGGAGTCCTGCGAGAA-3′ and 5′-GCTTCGAAGTCGTAGTCCTCAA-3′;

*E(spl)m6*: probe #55, 5′-TCCAACTAGTCCAAAGGATGC-3′ and 5′-AACCATCGAGGGTCTCCAA-3′;

*E(spl)m7*: probe#66, 5′-AGCGACAACGAGTCTCTGCT-3′ and 5′-TTACCAGGGACGCCACAC-3′;

*E(spl)m8*: probe#70, 5′-AGCAATTCCACGAAGCACA-3′ and 5′-GAGGAGCAGTCCATCGAGTT-3′;

*E(spl)mgamma*: probe#153, 5′-TCGATGTGACCAAGATGGAG-3′ and 5′-TATCTACCAGGGACGCCAGA-3′;

*E(spl)mdelta*: probe#60, 5′-CATTGTAATTTATTTCATCAACTTTGC-3′ and 5′-TTAATGAGGCTAAGTGGAAGCTC-3′;

*RpL19*: probe #128, 5′-GAGCGTATTGCCACCAGGA-3′ and 5′- CGATCTCGTCCTCCTTAGCA-3′;

*RpL32*: probe #117, 5′-CGGATCGATATGCTAAGCTGT-3′and 5′- CGACGCACTCTGTTGTCG-3′.

## Supporting Information

S1 Fig *E(spl)m7-lacZ* is expressed in both niche cap cells and GSCs. (A and B) One-week (w)-old wild-type (wt) germaria with ß-gal (red, *E(spl)m7-lacZ*, a *N* reporter), 1B1 (green, fusomes), Lam C (green, cap cell nuclear envelopes), and DAPI (gray, DNA) labels. B shows a germarium that was not stained with anti-ß-gal antibodies (Ab), as a negative control for the specificity of ß-gal signals detected in the GSCs shown in A. A′ and B′ show the *E(spl)m7-lacZ* (gray) channel only. Wt GSCs are outlined by dashed lines. Scale bar, 5 µm.
(TIF)

S2 Fig *E(spl)m7-lacZ* expression is decreased in GSCs with defective Notch signaling. (A and B) One-week (w)-old control (ctrl) (A) and *N*-knock down germaria (B) with 1B1 (green, fusomes), LamC (green, cap cells nuclear envelopes), and *E(spl)m7-LacZ* (gray, *N* reporter) labels. Dashed circles mark GSCs; arrow heads indicate niche cap cells. A′ and B′ show the *E(spl)m7-lacZ* (gray) channel only. Scale bar: 10 µm. (C and D) Average (avg.) intensity of ß-gal signals in cap cells (C) and GSCs (D). The number of cells analyzed is shown above each bar. ***, *P*<0.001. Error bars, mean ± SEM. The genotype of ctrl in A, C, and D is *tub-Gal80^ts^*/+; *nos-gal4-vp16 E(spl)m7-LacZ/+*.
(TIF)

S3 Fig Apoptotic germ cells are not observed in N and *fng* mutant mosaic germaria. (A) Four-day (D)-old wild-type (wt) starved germarium; (B) 1-week (w)-old *FRT19A* control; (C) 1-w-old *N^55e11^* mutant; and (D) 3-w-old *fng^[L73]^* mutant mosaic germaria. Germaria in B and C express GFP, and are labeled with ApopTag (red, apoptotic cells), 1B1 (blue, fusomes), LamC (blue, terminal filament and cap cell nuclear envelopes), and/or ß-gal (green, in D). The wt GSC is outlined by a solid line; GFP or ß-gal negative GSCs are outlined by dashed lines. Arrowheads indicate apoptotic somatic cells. Scale bar: 10 µm. (C′) A second *N^55e11^* mutant GSC on a different focal plane. (C″) Apoptotic somatic cells in a single channel. As a positive control (A), apoptotic cells were induced at the 2a/2b junction by starving the female on sugar and water media for two days before dissection [67].
(TIF)

S4 Fig *N^55e11^* and *fng^[L73]^* exhibit decreased Notch signaling, while *N^AXE2^* mutant GSCs exhibit increased Notch signaling. (A–E) One-week (W)-old germaria with GFP (green, wild-type (w) cells), ß-gal (gray, *E(spl)m7-lacZ*, a *N* reporter), 1B1 (gray, fusomes), and LamC (gray, cap cell nuclear envelopes) labels. Wt GSCs are outlined by solid lines, and control or mutant GSCs (GFP-negative) are outlined by dashed lines. Asterisks indicate cap cells. A′-E′ are show the *E(spl)m7-lacZ* channel only. (F) Average fold changes (FC) of *E(spl)m7-lacZ* expression in GFP^−^ GSCs as compared to neighboring wt GSCs within the same niche at 1w after clone induction. Numbers of germaria analyzed are shown above each bracket. *, *P*<0.05; ***, *P*<0.001. Error bar, mean ± SEM. Scale bars, 5 µm. The genotypes of the ctrl in A and F are *hs-flpneoFRT19A/ubiGFPFRT19A*, and in D and F are *hs-flp/+; neoFRT80B/ubiGFPFRT80B*.
(TIF)

S5 Fig E-cadherin expression and niche contact area are increased at the *fng^[L73]^* mutant GSC-niche junction. (A) Two-week (w)-old control (ctrl) and *fng^[L73]^* mutant mosaic germaria with ß-gal (green, wt cells), 1B1 (blue, fusomes), LamC (blue, cap cell nuclear envelopes), and E-cadherin (E-cad, red) labels. Wt GSCs are outlined by solid lines; ß-gal-negative (ß-gal ^−^) GSCs are outlined by dashed lines. Scale bar, 5 µm. A′, contact area between niche cap cells (green) and the GSCs shown in A. ß-gal-positive (ß-gal ^+^) GSCs are red and ß-gal^−^ GSCs are blue. Scale bar, 1 µm. (B) Average fold change (FC) of E-cad expression in ß-gal^−^ GSCs relative to that in ß-gal^+^ GSCs, in ctrl or *fng* mutant mosaic germaria at 1, 2, or 3w after clone induction. (C) Contact areas between niche cap cells and ß-gal^+^ GSCs or ß-gal^−^ GSCs at 1, 2, and 3w after clone induction. The numbers of analyzed germaria are shown above each bracket. *, *P*<0.05; **, *P*<0.01. Error bar, mean ± SEM. The genotype of control germaria is *hs-flp/+; arm-LacZFRT80B/ubiGFPFRT80B*.
(TIF)

S6 Fig *Su(H)Gbe-lacZ* expression is increased in GSCs with age. (A and B) One-week (W)-old (A) and 7-w-old germaria (B) with ß-gal (gray, *Su(H)Gbe-lacZ*, a Notch signaling reporter), 1B1 (green, GSC fusomes), and LamC (green, cap cell nuclear envelopes) labels. A′ and B′ show the *Su(H)Gbe-lacZ* channel only. Dashed circles mark GSCs; asterisks indicate niche cap cells. Scale bar: 5 µm. (C and D) Average (Avg.) ß-gal signals per cap cell (C) and GSC (D) in 1- and 5-W-old germaria. The number of cells analyzed is shown above each bar. *, *P*<0.05; ***, *P*<0.001. Error bar, mean ± SEM.
(TIF)

S7 Fig Expression levels of *Dl* and *Ser* reporters are not increased in germaria with age. (A–D) Germaria with 1B1 (green, fusomes), LamC (green, terminal filament (TF) and cap cell nuclear envelopes), *Dl-lacZ* (red, *Dl* reporter) (in A and B), and *Ser-lacZ* (red, *Ser* reporter) (C and D) labels at 1 week (W) (A and C) and 5W (B and D) after eclosion. *Dl-lacZ* is expressed in a subset of niche cap cells and the germline, while *Ser-lacZ* exhibits strong expression in the TF, but weak expression in the germline. Dashed circles mark GSCs; asterisks indicate niche cap cells. Solid lines in C and D indicate the edge of germaria. A′ and B′ show the *Dl-lacZ* channel only, and C′ and D′ show the *Ser-lacZ* channel only. Scale bar: 10 µm.
(TIF)

S8 Fig Sxl controls GSC differentiation. (A and B) One-week (w)-old *sxl^RNAi^*-knock down germaria with 1B1 (green, fusomes) and LamC (green, cap cell nuclear envelopes) labels. Approximately 20% of *sxl^RNAi^*-knock down germaria carried tumorous GSCs containing round fusomes, as shown in B. (C and D) One-week (w)-old *sxl^f2^* mutant mosaic germaria with GFP (green, wild-type cells), 1B1 (red, fusomes), and LamC (red, cap cell nuclear envelopes) labels. The *sxl^f2^* mutant GSCs/cystoblasts failed to undergo differentiation, and thus accumulated in germaria. Dashed circles mark GSCs residing within the niche. Scale bar: 10 µm.
(TIF)

S9 Fig Expression of Sxl is decreased in ovaries with age. (A and B) Western blotting analysis of Sxl expression in one- and seven-week (W)-old wild-type ovaries (A) and in one- and five-W-old GSCs isolated from *bam* mutant females (B). Molecular weight *markers* are *shown* at the left of the blots. (A′ and B′) Relative expression of proteins in 7-W-old ovaries as compared to that in 1-W-old ovaries (A′), and in 5-W-old GSCs as compared to that in 1-W-old GSCs (B′). *, *P*<0.05.
(TIF)

S10 Fig Manipulation of Notch signaling in GSCs alters their maintenance with age. (A) 1-week (W) -old *mat-GAL4-*driven *UASp-mCD8-GFP* germarium with GFP (green), 1B1 (red, fusomes), and LamC (red, cap cell nuclear envelopes) labels. Scale bar, 10 µm. (B) Relative percentage of GSCs remaining in the germaria of the indicated genotypes at different ages. Flies carrying GFP or *GFP^RNAi^* driven by *mat-GAL4* were used as controls. N^NICD^ was used to increase Notch signaling; RNAi lines were used to suppress the expression of *N*. The *GAL4* control (ctrl) was obtained by crossing the *GAL4* line with the *w^1118^* strain. Line graphs show the maintenance rates of GSCs with age (N, just eclosed; weeks, shown on the x axis). GSC maintenance rates for each genotype were determined by normalizing the average numbers of GSCs at each time point to that in newly eclosed flies. The maintenance rates of both *N^NICD^*-overexpressing and *N^RNAi^*-knock down GSCs were significantly different from those of all the controls (*P*<0.05), as determined by Wilcoxon Signed-Rank test. Bar graphs indicate the distribution of germaria with 0, 1, 2, or 3 or more GSCs at the indicated weeks. The number of germaria analyzed is shown above each bar.
(TIF)

S11 Fig GSCs in the niche express different levels of *E(spl)m7-lacZ*. (A and B) Seven-week (w)-old *yw* germaria with ß-gal (gray, E(spl)m7-lacZ, a Notch signaling reporter), 1B1 (green, fusomes), and LamC (green, cap cell nuclear envelopes) labels. GSCs are outlined by dashed lines. A and A′ show the same germarium, but on a different focal plane. The asterisk indicates the position of out-of-focus cap cells. Scale bar: 10 µm.
(TIF)

S12 Fig Similar levels of Notch signaling are detected in GSCs and their progeny in the testis. One-week (W)-old wild-type testis with 1B1 (red, fusomes) and ß-gal (green, *E(spl)m7-lacZ*, a Notch reporter) labels. Asterisks represent GSCs; dashed circles outline niche hub cells. Scale bar, 20 µm.
(TIF)

S13 Fig Sxl does not control Notch activation in niche cap cells, and disruption of *sxl, N*, or *Dl* in niche cap cells does not affect Notch activation in GSCs. (A) One-week (W)-old wild-type, *sxl*, *N*, and *Dl* knock down germaria with 1B1 (green, fusomes), LamC (green, nuclear envelopes of cap cells), ß-gal (red, *E(spl)m7-lacZ*, a Notch reporter), and DAPI (blue, DNA) labels. Asterisks mark cap cells; dashed circles outline GSCs. Scale bar, 5 µm. (B) Average (avg.) *E(spl)m7-lacZ* expression levels per GSC (B) and per cap cell (B′) in flies of the indicated genotypes. Total GSCs or cap cells analyzed are shown above each bar. ***, *P*<0.001; error bar, SEM. The genotype of the ctrl in A and B is *tub-Gal80ts*/+; *bab1-GAL4/E(spl)m7-LacZ*.
(TIF)

S14 Fig Notch signaling is not decreased in GSCs with defective insulin signaling. (A and B) One-week (W)-old heterozygous control (hetero. ctrl.) and *dinr^339^/dinr^E19^* mutant germaria with LamC (green, nuclear envelopes of niche cells), 1B1 (green, fusomes), and ß-gal (gray, *E(spl)m7-lacZ*, a Notch reporter) labels. A′ and B′ show the *E(spl)m7-lacZ* channel only. Asterisks indicate niche cap cells; dashed circles outline GSCs. Scale bar, 5 µm. (C) Average (avg.) intensity of ß-gal in control and *dinr^339^/dinr^E19^* mutant GSCs one week after eclosion. Total numbers of GSCs analyzed are shown above each bar. The genotype of control flies shown in **A** and **C** is dinr^339^/+.
(TIF)

S15 Fig **The** *sxl^RNAi^* **and** *shg^RNAi^* **constructs efficiently disrupt Sxl and E-cadherin in the germline.** (A and B) One-week (w)-old ctrl (A) and *sxl*-knock down germaria (B) with Vasa (green, germ cells), Sxl (gray), and DAPI (blue, DNA) labels. A′ and B′ show the Sxl channel only. (C and D) Three-dimensional reconstructed images of one-week-old ctrl (C) and *shg*-knock down germaria (D) with LamC (green, nuclear envelopes of cap cells), 1B1 (green, fusomes), and E-cadherin (E-cad, gray) labels. Dashed lines indicate the position of GSCs. Scale bars: 5 µm. (E) Average (avg.) E-cad intensity at the junction between GSCs and their niche in 1-w-old ctrl and *nos>shg^RNAi^* germaria. Total GSCs analyzed are shown above each bar. ***, *P*<0.001; error bar, SEM. The genotype of the ctrl is *tub-Gal80ts*/+; *nos-gal4-vp16/+* in A and *tub-Gal80ts/UAS-mCD8-GFP; nos-gal4-vp16/+* in C and E.
(TIF)

S1 Table Notch signaling controls GSC competition for niche occupancy via E-cadherin.
(DOCX)
