# Correction: Notch Signaling Mediates the Age-Associated Decrease in Adhesion of Germline Stem Cells to the Niche

**DOI:** 10.1371/journal.pgen.1005766

**Published:** 2015-12-18

**Authors:** Chen-Yuan Tseng, Shih-Han Kao, Chih-Ling Wan, Yueh Cho, Shu-Yun Tung, Hwei-Jan Hsu

The affiliation for the 6th author is incomplete. Hwei-Jan Hsu is affiliated with:

1. Institute of Cellular and Organismic Biology, Academia Sinica, Taipei, Taiwan, and

2. Graduate Institute of Life Sciences, National Defense Medical Center, Taipei, Taiwan
