# Supplementary figures and images for: Notch Signaling Mediates the Age-Associated Decrease in Adhesion of Germline Stem Cells to the Niche
Source: PLoS Genet. 2014 Dec 18;10(12):e1004888. doi: 10.1371/journal.pgen.1004888 (PMC4270478; doi:10.1371/journal.pgen.1004888)

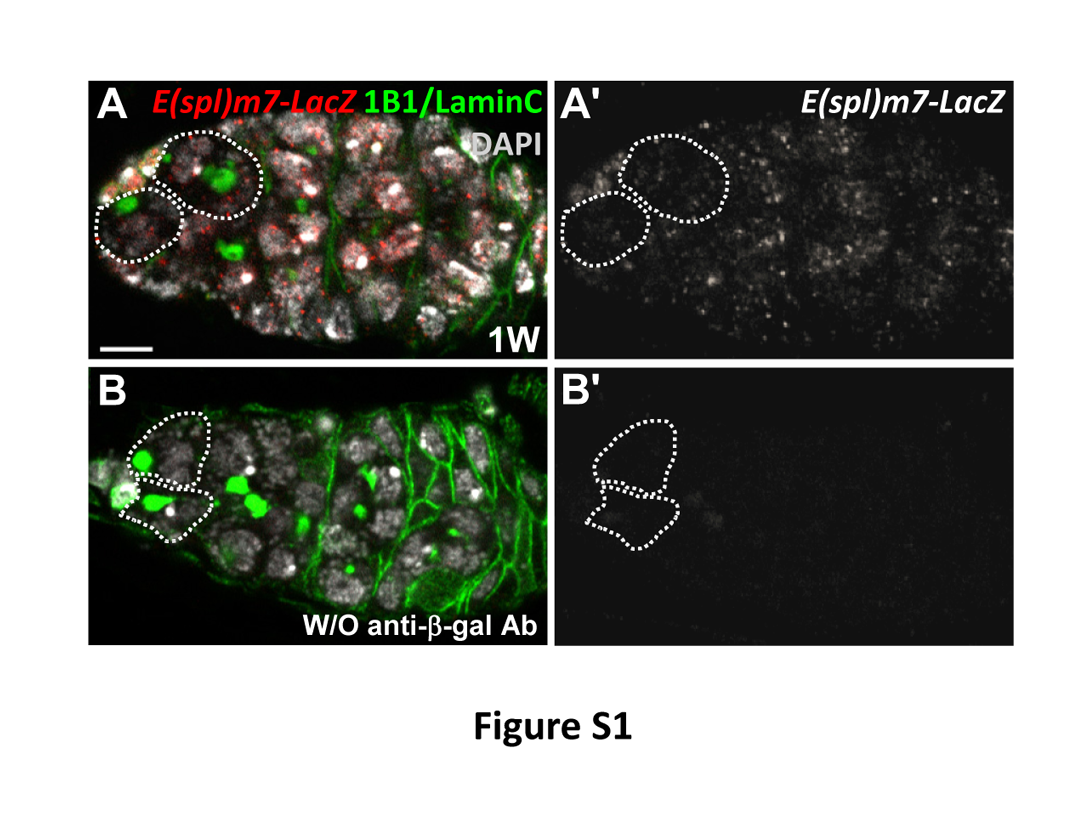

Supplement: S1 Fig — E(spl)m7-lacZ is expressed in both niche cap cells and GSCs. (A and B) One-week (w)-old wild-type (wt) germaria with ß-gal (red, E(spl)m7-lacZ, a N reporter), 1B1 (green, fusomes), Lam C (green, cap cell nuclear envelopes), and DAPI (gray, DNA) labels. B shows a germarium that was not stained with anti-ß-gal antibodies (Ab), as a negative control for the specificity of ß-gal signals detected in the GSCs shown in A. A′ and B′ show the E(spl)m7-lacZ (gray) channel only. Wt GSCs are outlined by dashed lines. Scale bar, 5 µm. (TIF) [file pgen.1004888.s001.tif]

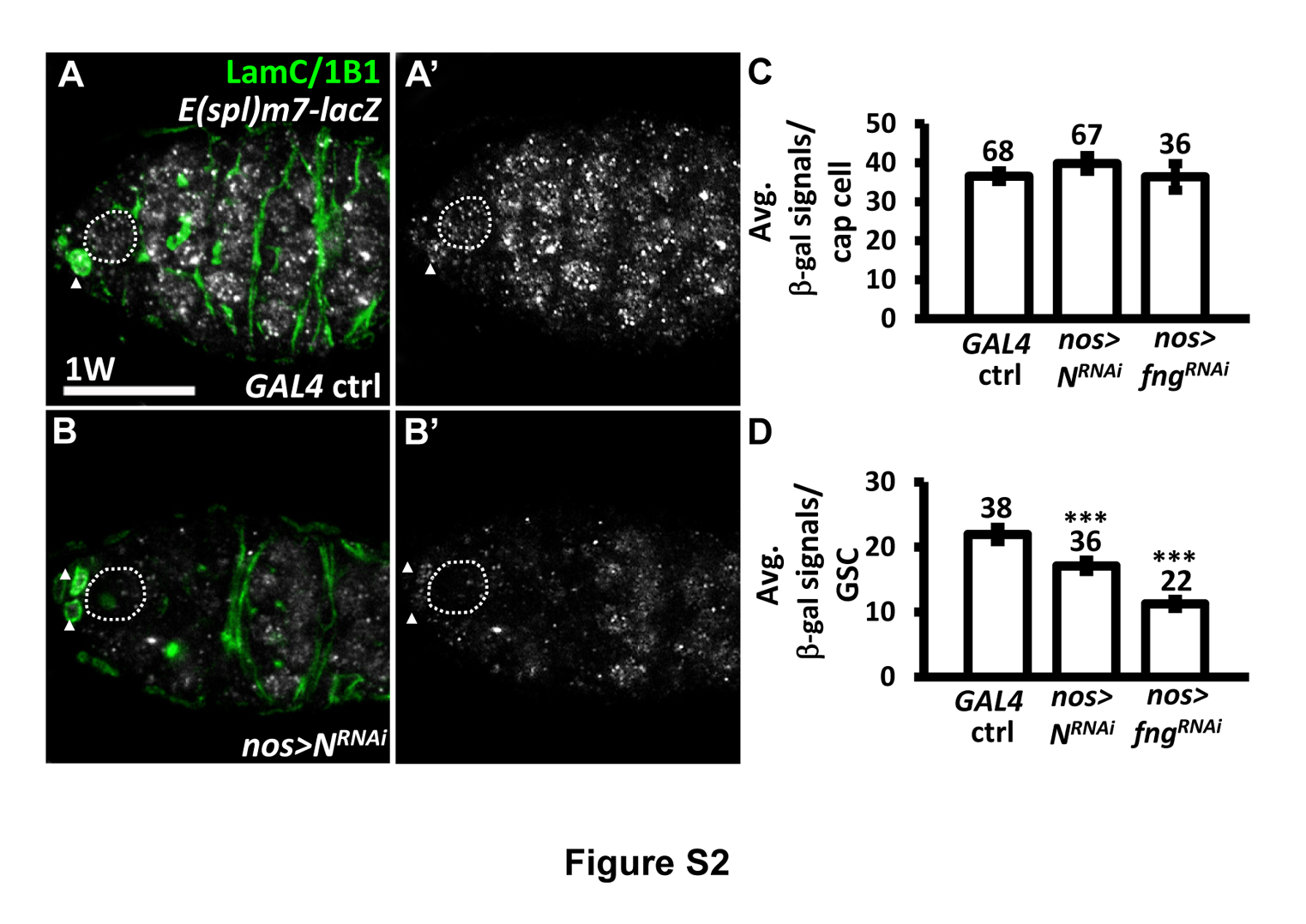

Supplement: S2 Fig — E(spl)m7-lacZ expression is decreased in GSCs with defective Notch signaling. (A and B) One-week (w)-old control (ctrl) (A) and N-knock down germaria (B) with 1B1 (green, fusomes), LamC (green, cap cells nuclear envelopes), and E(spl)m7-LacZ (gray, N reporter) labels. Dashed circles mark GSCs; arrow heads indicate niche cap cells. A′ and B′ show the E(spl)m7-lacZ (gray) channel only. Scale bar: 10 µm. (C and D) Average (avg.) intensity of ß-gal signals in cap cells (C) and GSCs (D). The number of cells analyzed is shown above each bar. ***, P<0.001. Error bars, mean ± SEM. The genotype of ctrl in A, C, and D is tub-Gal80ts/+; nos-gal4-vp16 E(spl)m7-LacZ/+. (TIF) [file pgen.1004888.s002.tif]

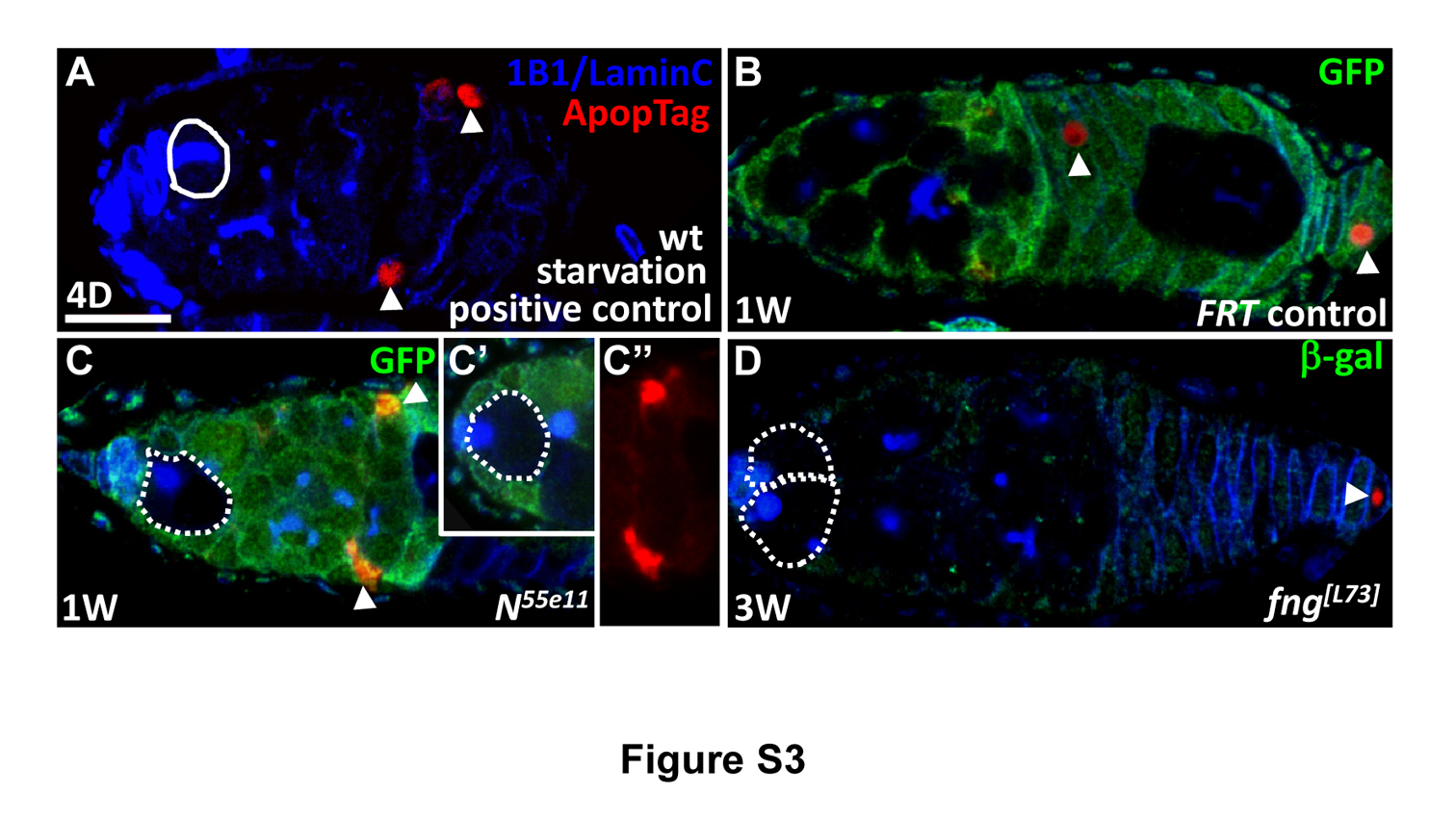

Supplement: S3 Fig — Apoptotic germ cells are not observed in N and fng mutant mosaic germaria. (A) Four-day (D)-old wild-type (wt) starved germarium; (B) 1-week (w)-old FRT19A control; (C) 1-w-old N55e11 mutant; and (D) 3-w-old fng[L73] mutant mosaic germaria. Germaria in B and C express GFP, and are labeled with ApopTag (red, apoptotic cells), 1B1 (blue, fusomes), LamC (blue, terminal filament and cap cell nuclear envelopes), and/or ß-gal (green, in D). The wt GSC is outlined by a solid line; GFP or ß-gal negative GSCs are outlined by dashed lines. Arrowheads indicate apoptotic somatic cells. Scale bar: 10 µm. (C′) A second N55e11 mutant GSC on a different focal plane. (C″) Apoptotic somatic cells in a single channel. As a positive control (A), apoptotic cells were induced at the 2a/2b junction by starving the female on sugar and water media for two days before dissection [67]. (TIF) [file pgen.1004888.s003.tif]

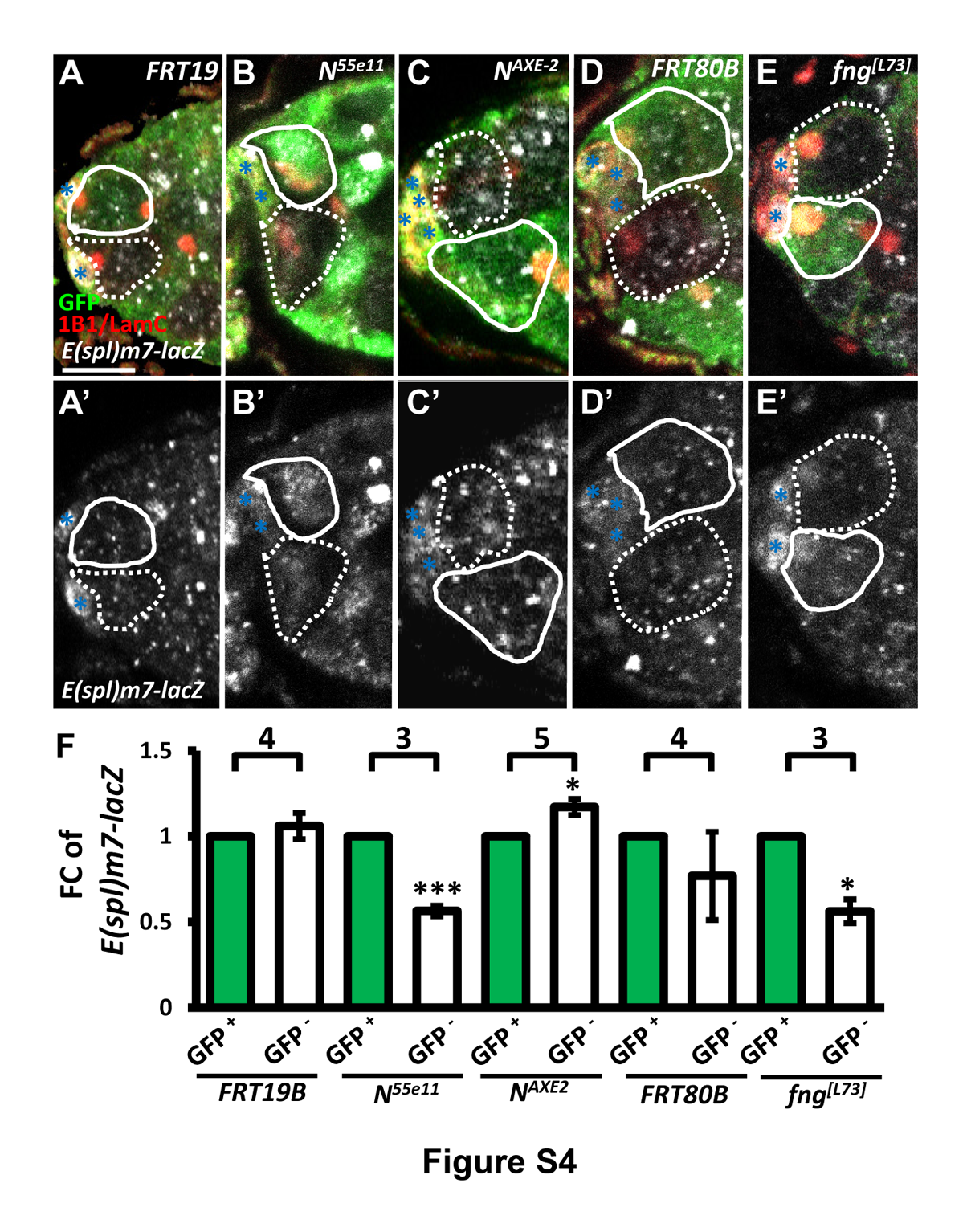

Supplement: S4 Fig — N55e11 and fng[L73] exhibit decreased Notch signaling, while NAXE2 mutant GSCs exhibit increased Notch signaling. (A–E) One-week (W)-old germaria with GFP (green, wild-type (w) cells), ß-gal (gray, E(spl)m7-lacZ, a N reporter), 1B1 (gray, fusomes), and LamC (gray, cap cell nuclear envelopes) labels. Wt GSCs are outlined by solid lines, and control or mutant GSCs (GFP-negative) are outlined by dashed lines. Asterisks indicate cap cells. A′-E′ are show the E(spl)m7-lacZ channel only. (F) Average fold changes (FC) of E(spl)m7-lacZ expression in GFP− GSCs as compared to neighboring wt GSCs within the same niche at 1w after clone induction. Numbers of germaria analyzed are shown above each bracket. *, P<0.05; ***, P<0.001. Error bar, mean ± SEM. Scale bars, 5 µm. The genotypes of the ctrl in A and F are hs-flpneoFRT19A/ubiGFPFRT19A, and in D and F are hs-flp/+; neoFRT80B/ubiGFPFRT80B. (TIF) [file pgen.1004888.s004.tif]

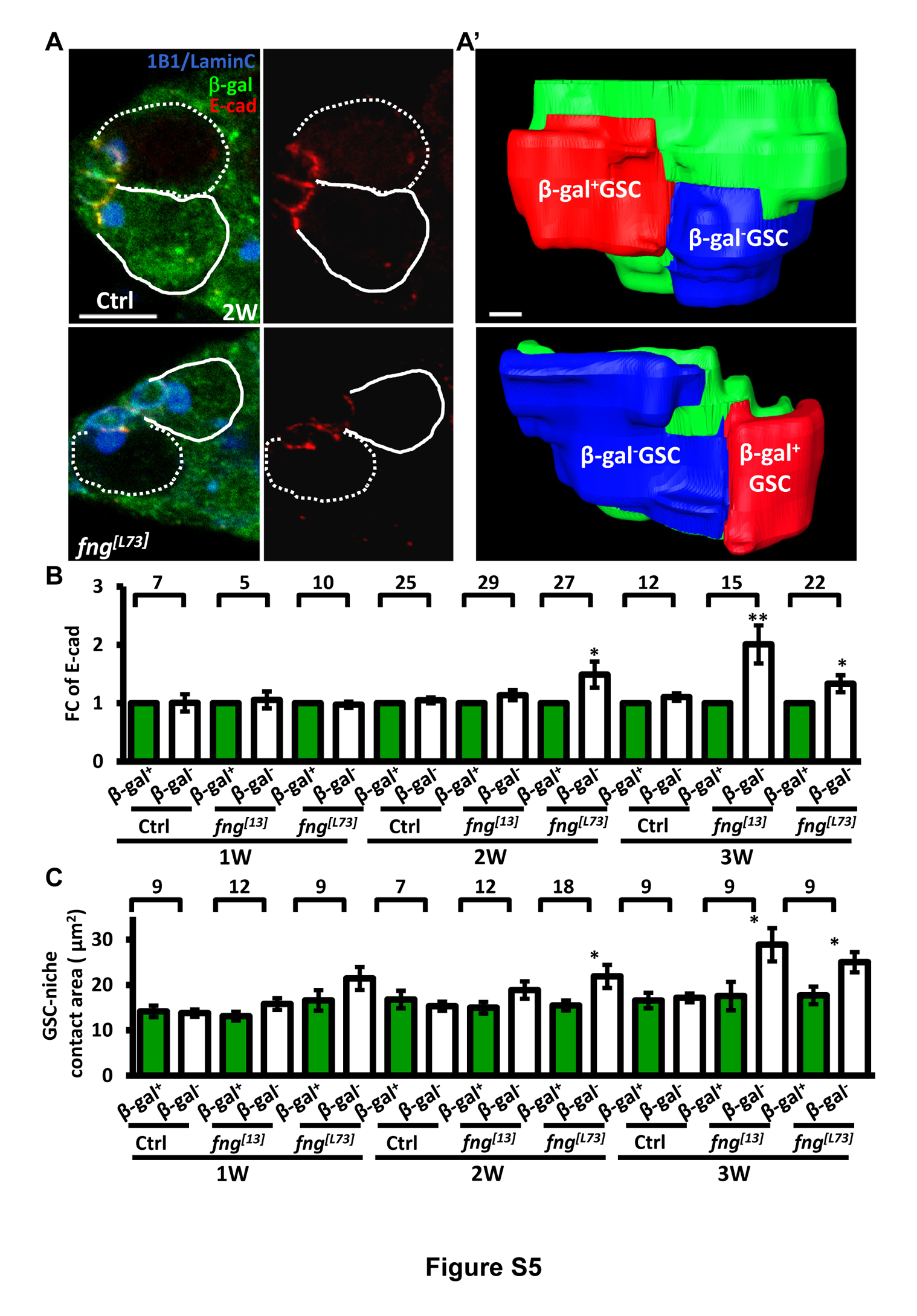

Supplement: S5 Fig — E-cadherin expression and niche contact area are increased at the fng[L73] mutant GSC-niche junction. (A) Two-week (w)-old control (ctrl) and fng[L73] mutant mosaic germaria with ß-gal (green, wt cells), 1B1 (blue, fusomes), LamC (blue, cap cell nuclear envelopes), and E-cadherin (E-cad, red) labels. Wt GSCs are outlined by solid lines; ß-gal-negative (ß-gal −) GSCs are outlined by dashed lines. Scale bar, 5 µm. A′, contact area between niche cap cells (green) and the GSCs shown in A. ß-gal-positive (ß-gal +) GSCs are red and ß-gal− GSCs are blue. Scale bar, 1 µm. (B) Average fold change (FC) of E-cad expression in ß-gal− GSCs relative to that in ß-gal+ GSCs, in ctrl or fng mutant mosaic germaria at 1, 2, or 3w after clone induction. (C) Contact areas between niche cap cells and ß-gal+ GSCs or ß-gal− GSCs at 1, 2, and 3w after clone induction. The numbers of analyzed germaria are shown above each bracket. *, P<0.05; **, P<0.01. Error bar, mean ± SEM. The genotype of control germaria is hs-flp/+; arm-LacZFRT80B/ubiGFPFRT80B. (TIF) [file pgen.1004888.s005.tif]

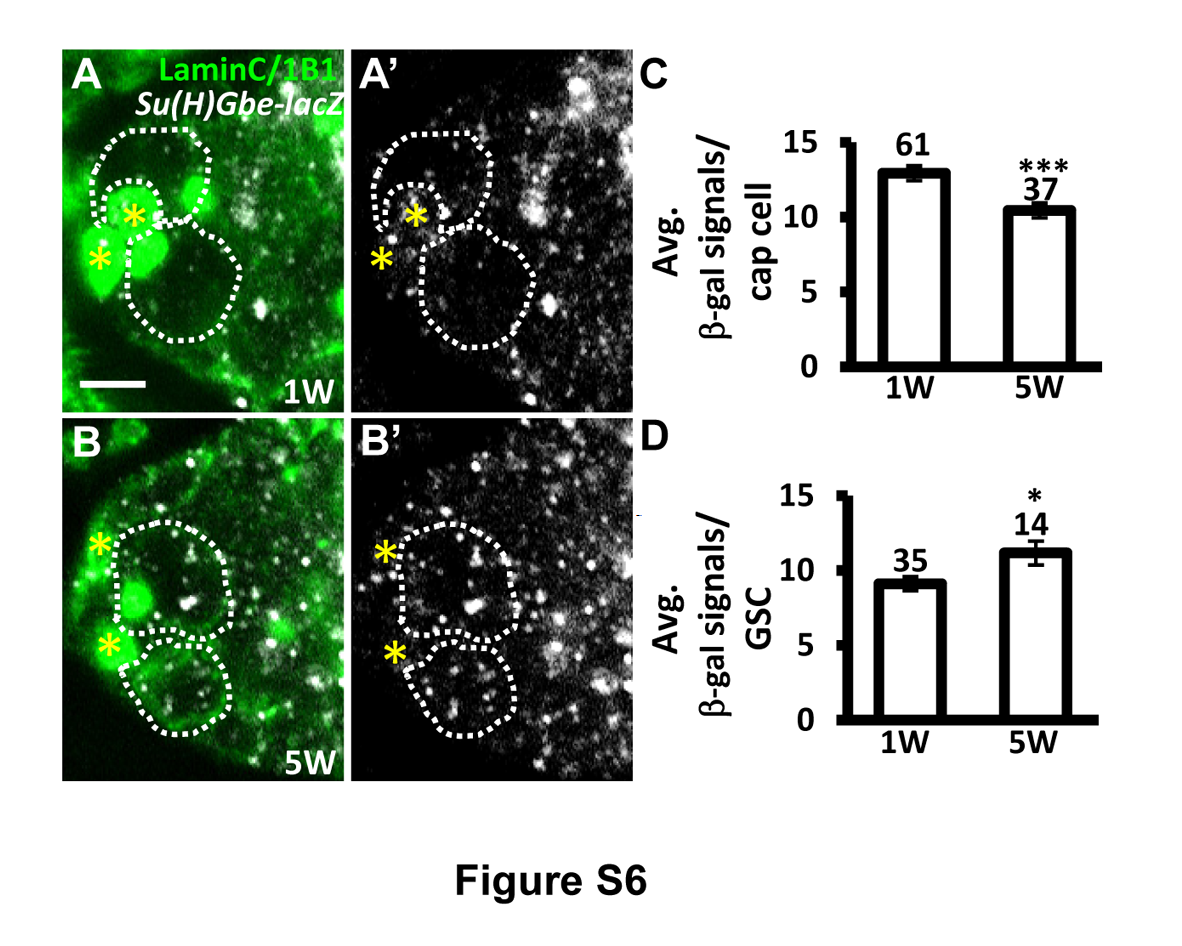

Supplement: S6 Fig — Su(H)Gbe-lacZ expression is increased in GSCs with age. (A and B) One-week (W)-old (A) and 7-w-old germaria (B) with ß-gal (gray, Su(H)Gbe-lacZ, a Notch signaling reporter), 1B1 (green, GSC fusomes), and LamC (green, cap cell nuclear envelopes) labels. A′ and B′ show the Su(H)Gbe-lacZ channel only. Dashed circles mark GSCs; asterisks indicate niche cap cells. Scale bar: 5 µm. (C and D) Average (Avg.) ß-gal signals per cap cell (C) and GSC (D) in 1- and 5-W-old germaria. The number of cells analyzed is shown above each bar. *, P<0.05; ***, P<0.001. Error bar, mean ± SEM. (TIF) [file pgen.1004888.s006.tif]

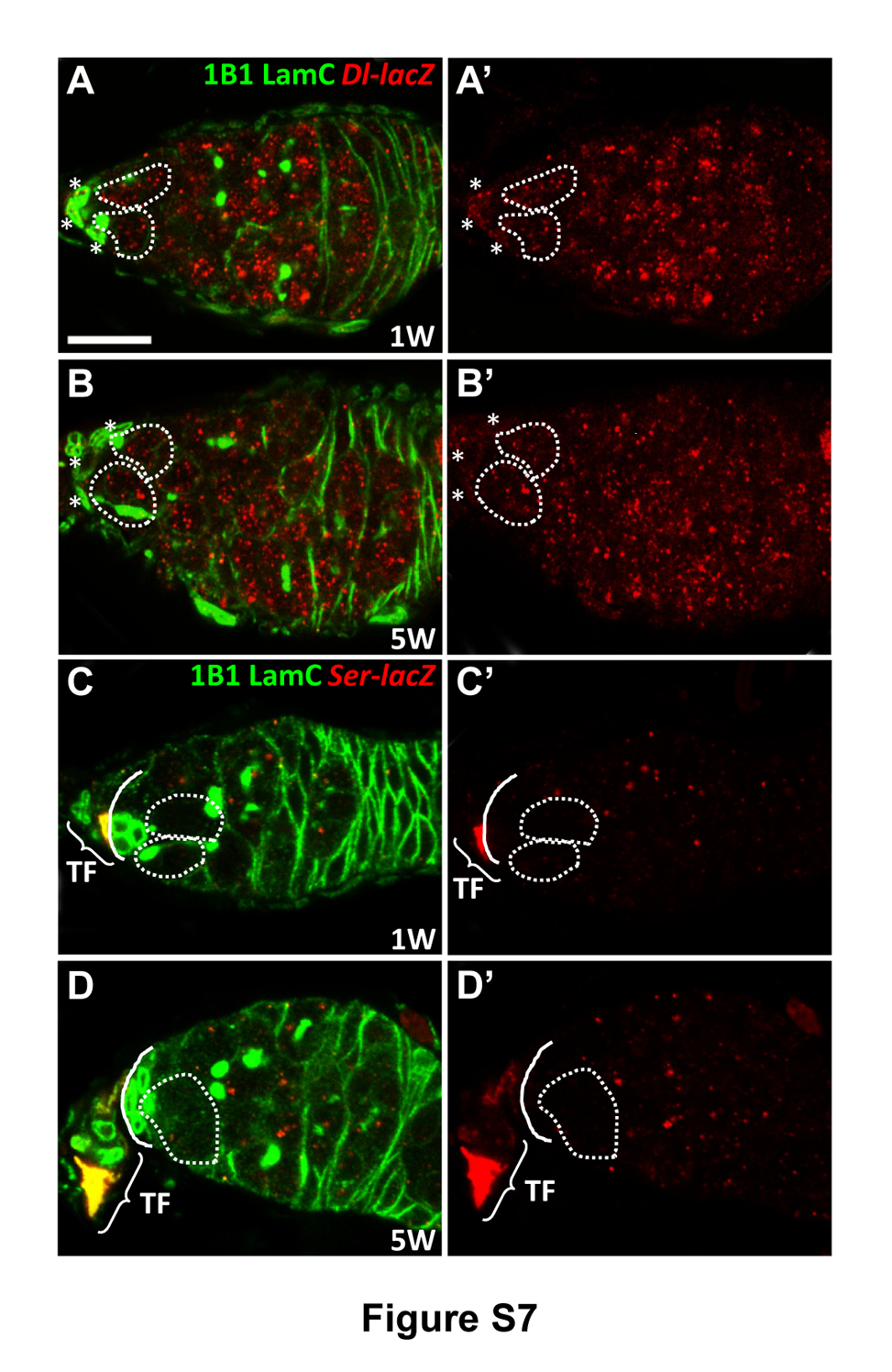

Supplement: S7 Fig — Expression levels of Dl and Ser reporters are not increased in germaria with age. (A–D) Germaria with 1B1 (green, fusomes), LamC (green, terminal filament (TF) and cap cell nuclear envelopes), Dl-lacZ (red, Dl reporter) (in A and B), and Ser-lacZ (red, Ser reporter) (C and D) labels at 1 week (W) (A and C) and 5W (B and D) after eclosion. Dl-lacZ is expressed in a subset of niche cap cells and the germline, while Ser-lacZ exhibits strong expression in the TF, but weak expression in the germline. Dashed circles mark GSCs; asterisks indicate niche cap cells. Solid lines in C and D indicate the edge of germaria. A′ and B′ show the Dl-lacZ channel only, and C′ and D′ show the Ser-lacZ channel only. Scale bar: 10 µm. (TIF) [file pgen.1004888.s007.tif]

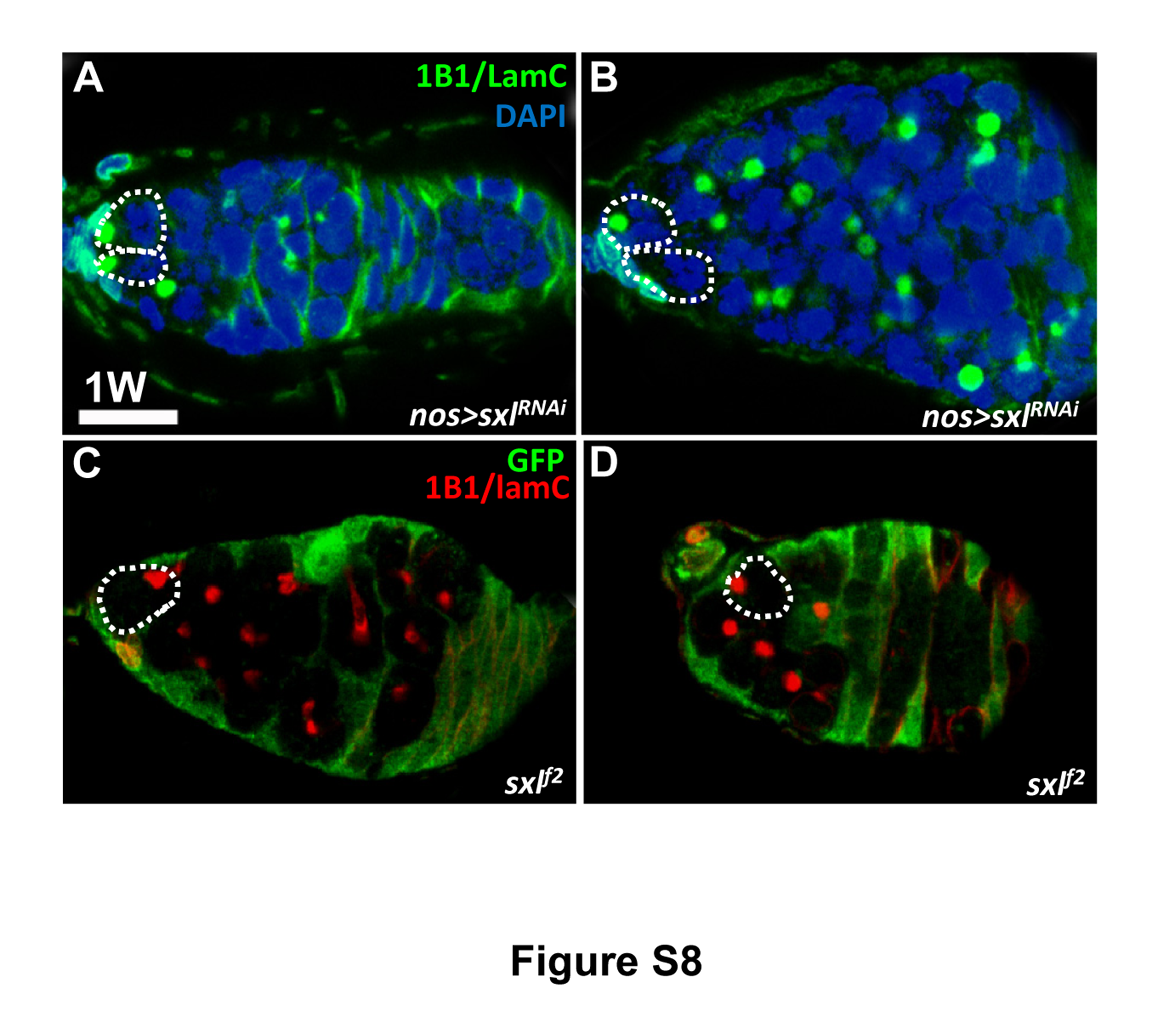

Supplement: S8 Fig — Sxl controls GSC differentiation. (A and B) One-week (w)-old sxlRNAi-knock down germaria with 1B1 (green, fusomes) and LamC (green, cap cell nuclear envelopes) labels. Approximately 20% of sxlRNAi-knock down germaria carried tumorous GSCs containing round fusomes, as shown in B. (C and D) One-week (w)-old sxlf2 mutant mosaic germaria with GFP (green, wild-type cells), 1B1 (red, fusomes), and LamC (red, cap cell nuclear envelopes) labels. The sxlf2 mutant GSCs/cystoblasts failed to undergo differentiation, and thus accumulated in germaria. Dashed circles mark GSCs residing within the niche. Scale bar: 10 µm. (TIF) [file pgen.1004888.s008.tif]

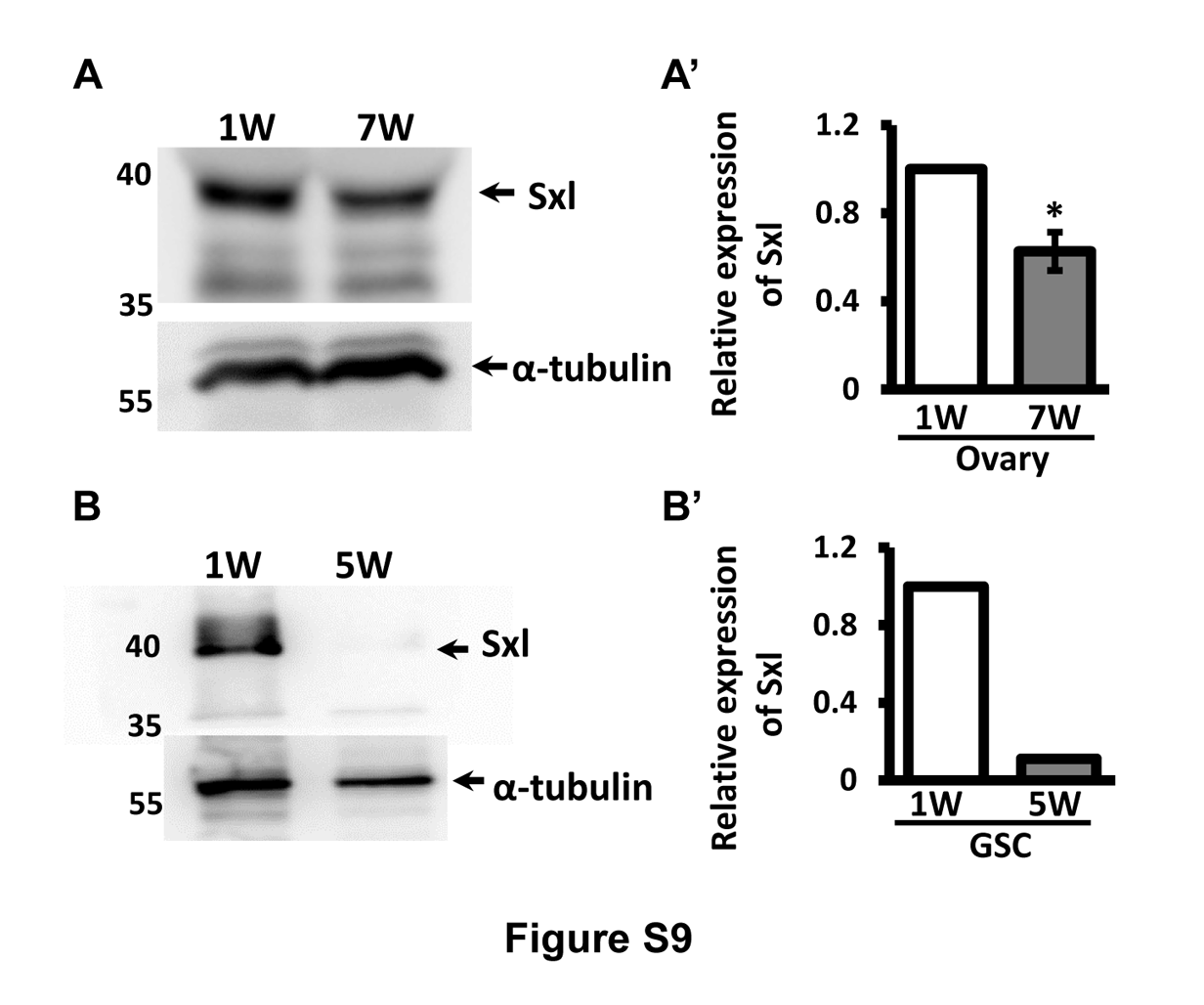

Supplement: S9 Fig — Expression of Sxl is decreased in ovaries with age. (A and B) Western blotting analysis of Sxl expression in one- and seven-week (W)-old wild-type ovaries (A) and in one- and five-W-old GSCs isolated from bam mutant females (B). Molecular weight markers are shown at the left of the blots. (A′ and B′) Relative expression of proteins in 7-W-old ovaries as compared to that in 1-W-old ovaries (A′), and in 5-W-old GSCs as compared to that in 1-W-old GSCs (B′). *, P<0.05. (TIF) [file pgen.1004888.s009.tif]

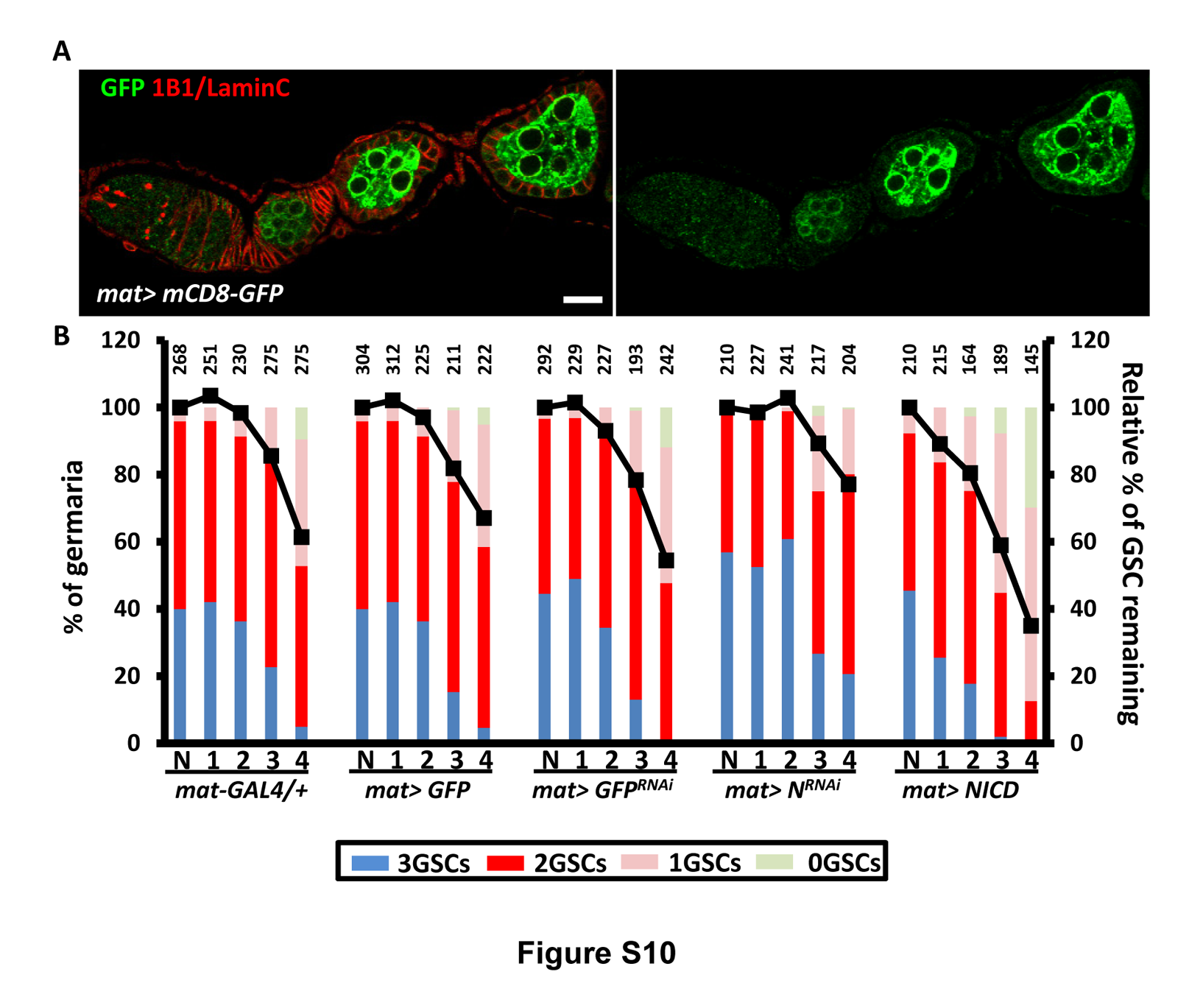

Supplement: S10 Fig — Manipulation of Notch signaling in GSCs alters their maintenance with age. (A) 1-week (W) -old mat-GAL4-driven UASp-mCD8-GFP germarium with GFP (green), 1B1 (red, fusomes), and LamC (red, cap cell nuclear envelopes) labels. Scale bar, 10 µm. (B) Relative percentage of GSCs remaining in the germaria of the indicated genotypes at different ages. Flies carrying GFP or GFPRNAi driven by mat-GAL4 were used as controls. NNICD was used to increase Notch signaling; RNAi lines were used to suppress the expression of N. The GAL4 control (ctrl) was obtained by crossing the GAL4 line with the w1118 strain. Line graphs show the maintenance rates of GSCs with age (N, just eclosed; weeks, shown on the x axis). GSC maintenance rates for each genotype were determined by normalizing the average numbers of GSCs at each time point to that in newly eclosed flies. The maintenance rates of both NNICD-overexpressing and NRNAi-knock down GSCs were significantly different from those of all the controls (P<0.05), as determined by Wilcoxon Signed-Rank test. Bar graphs indicate the distribution of germaria with 0, 1, 2, or 3 or more GSCs at the indicated weeks. The number of germaria analyzed is shown above each bar. (TIF) [file pgen.1004888.s010.tif]

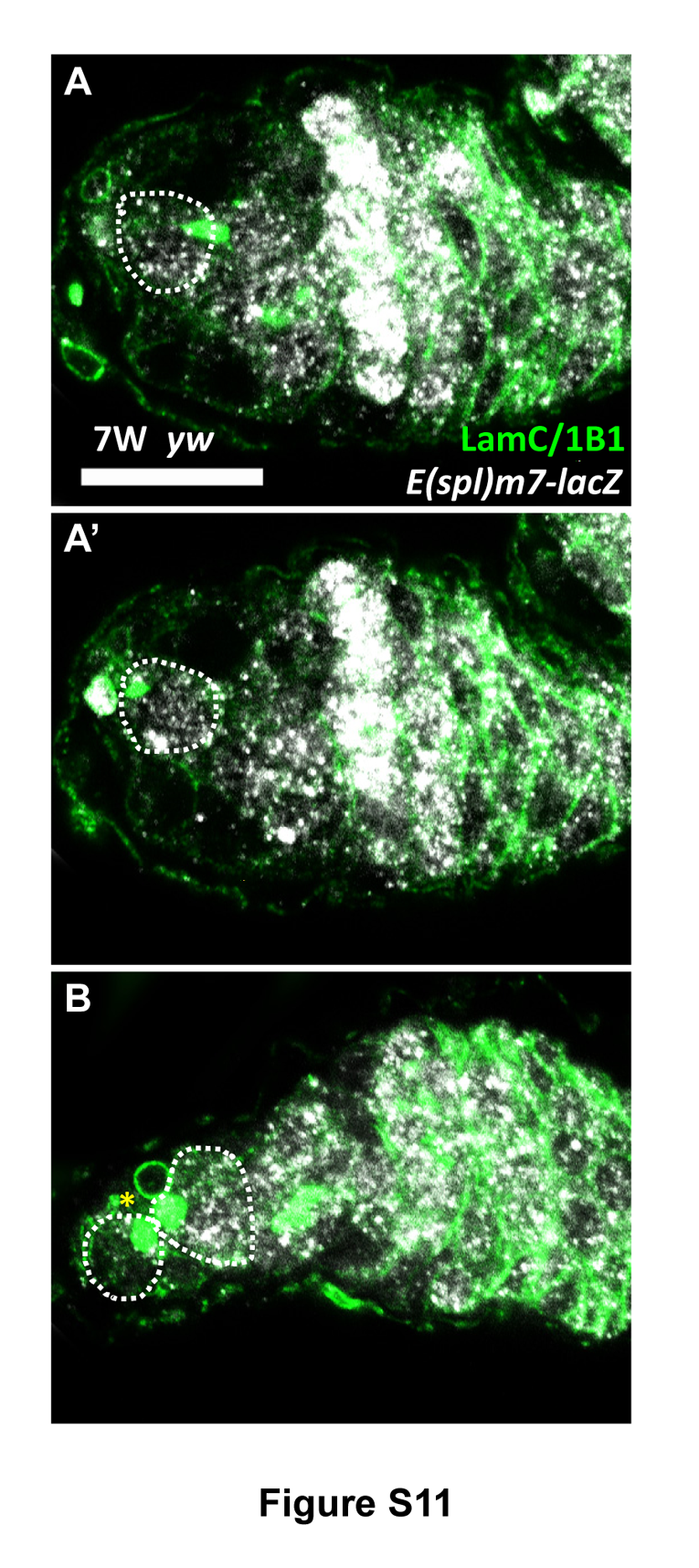

Supplement: S11 Fig — GSCs in the niche express different levels of E(spl)m7-lacZ. (A and B) Seven-week (w)-old yw germaria with ß-gal (gray, E(spl)m7-lacZ, a Notch signaling reporter), 1B1 (green, fusomes), and LamC (green, cap cell nuclear envelopes) labels. GSCs are outlined by dashed lines. A and A′ show the same germarium, but on a different focal plane. The asterisk indicates the position of out-of-focus cap cells. Scale bar: 10 µm. (TIF) [file pgen.1004888.s011.tif]

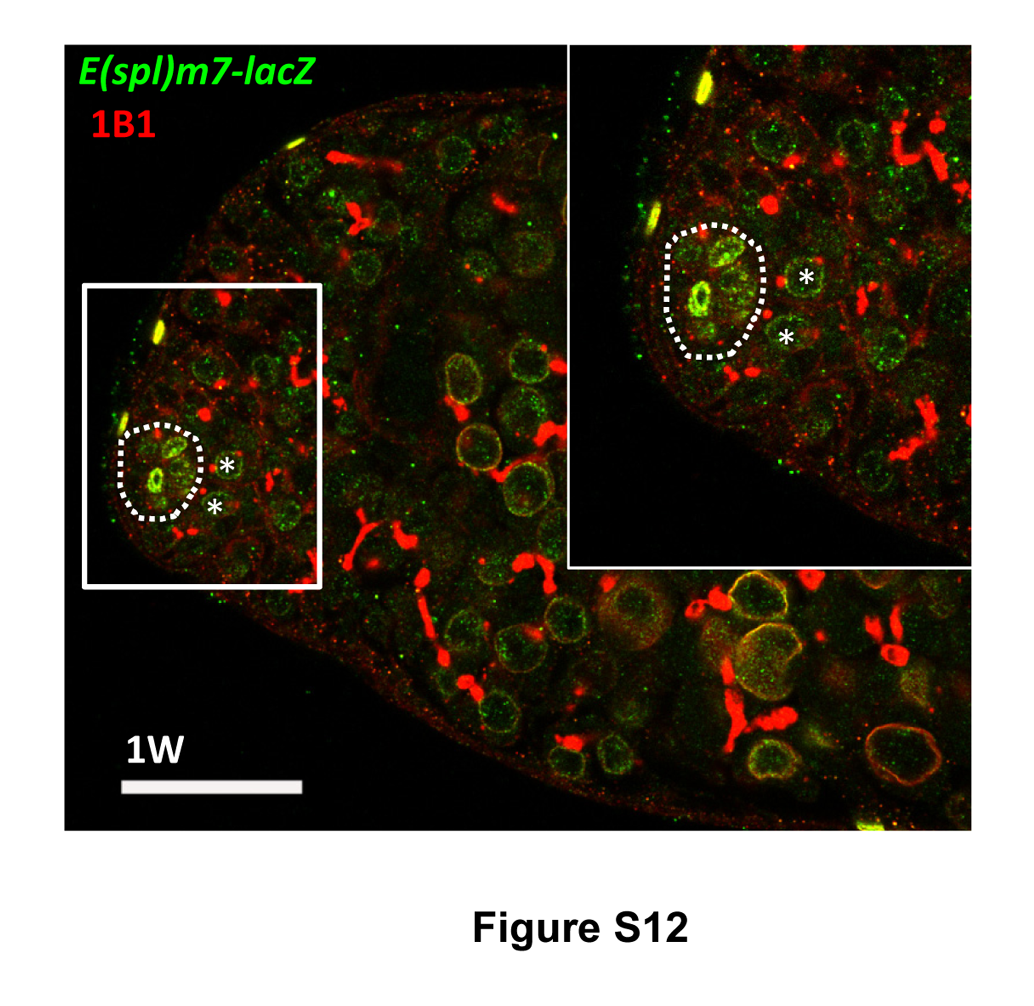

Supplement: S12 Fig — Similar levels of Notch signaling are detected in GSCs and their progeny in the testis. One-week (W)-old wild-type testis with 1B1 (red, fusomes) and ß-gal (green, E(spl)m7-lacZ, a Notch reporter) labels. Asterisks represent GSCs; dashed circles outline niche hub cells. Scale bar, 20 µm. (TIF) [file pgen.1004888.s012.tif]

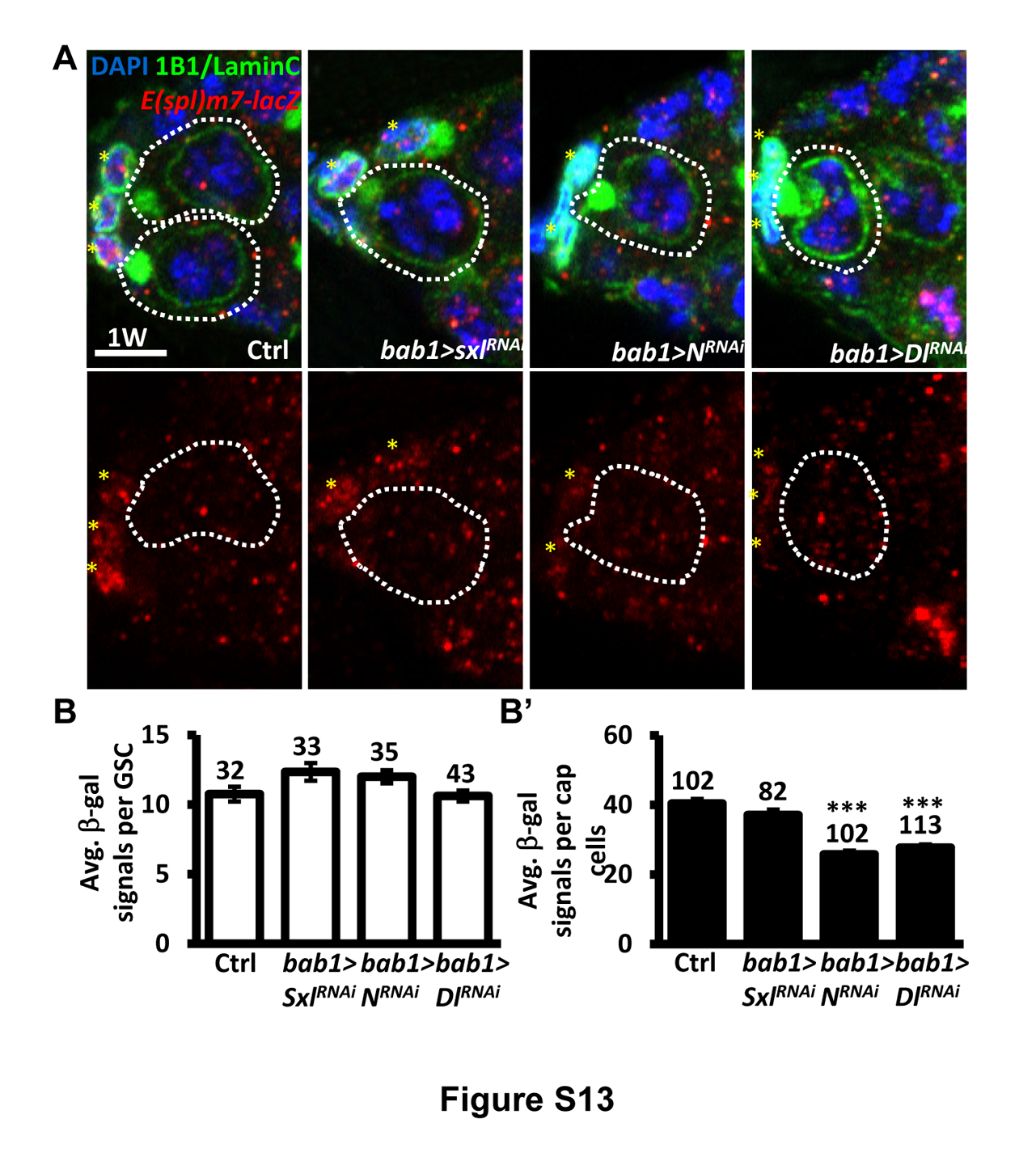

Supplement: S13 Fig — Sxl does not control Notch activation in niche cap cells, and disruption of sxl, N, or Dl in niche cap cells does not affect Notch activation in GSCs. (A) One-week (W)-old wild-type, sxl, N, and Dl knock down germaria with 1B1 (green, fusomes), LamC (green, nuclear envelopes of cap cells), ß-gal (red, E(spl)m7-lacZ, a Notch reporter), and DAPI (blue, DNA) labels. Asterisks mark cap cells; dashed circles outline GSCs. Scale bar, 5 µm. (B) Average (avg.) E(spl)m7-lacZ expression levels per GSC (B) and per cap cell (B′) in flies of the indicated genotypes. Total GSCs or cap cells analyzed are shown above each bar. ***, P<0.001; error bar, SEM. The genotype of the ctrl in A and B is tub-Gal80ts/+; bab1-GAL4/E(spl)m7-LacZ. (TIF) [file pgen.1004888.s013.tif]

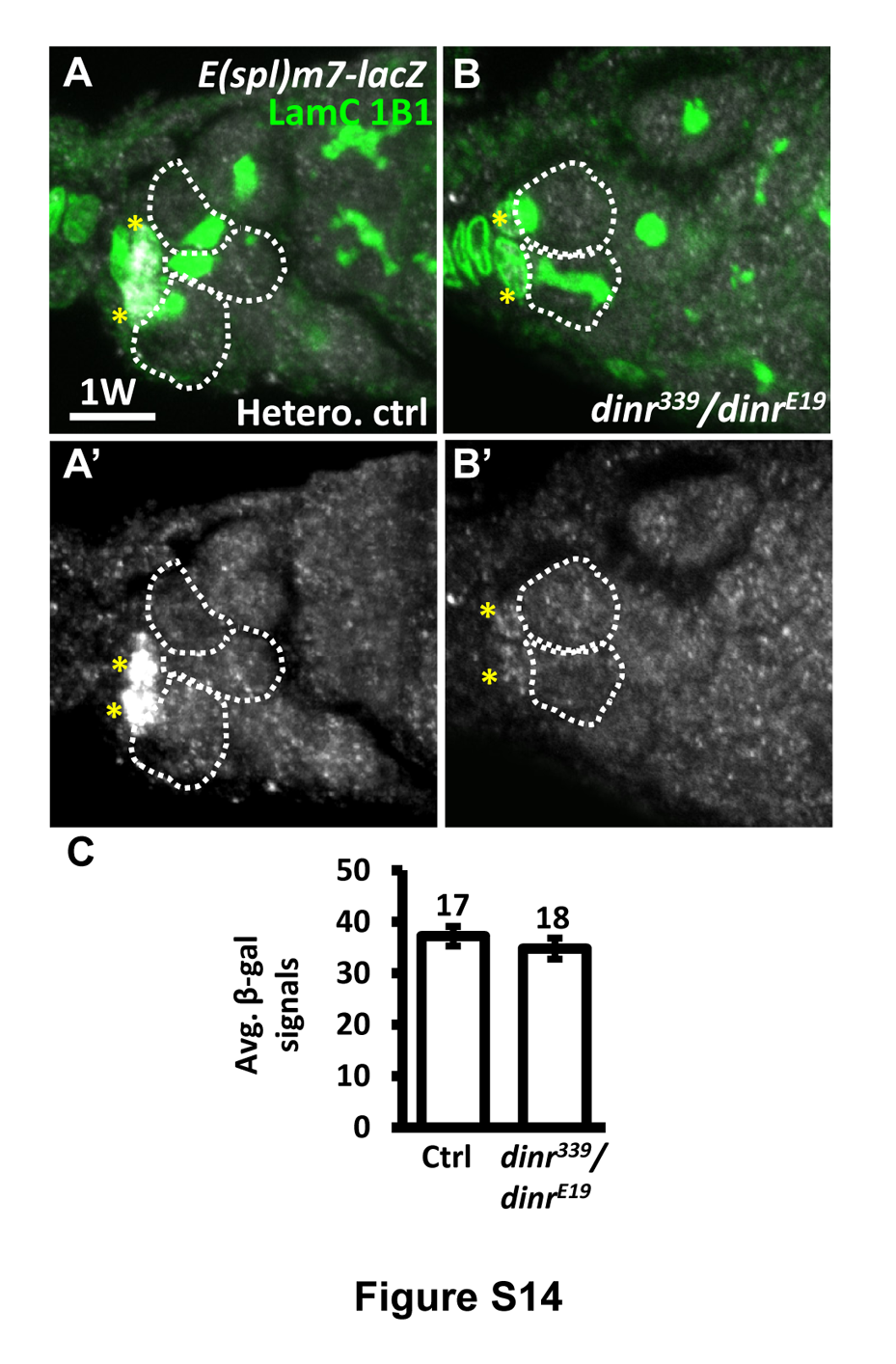

Supplement: S14 Fig — Notch signaling is not decreased in GSCs with defective insulin signaling. (A and B) One-week (W)-old heterozygous control (hetero. ctrl.) and dinr339/dinrE19 mutant germaria with LamC (green, nuclear envelopes of niche cells), 1B1 (green, fusomes), and ß-gal (gray, E(spl)m7-lacZ, a Notch reporter) labels. A′ and B′ show the E(spl)m7-lacZ channel only. Asterisks indicate niche cap cells; dashed circles outline GSCs. Scale bar, 5 µm. (C) Average (avg.) intensity of ß-gal in control and dinr339/dinrE19 mutant GSCs one week after eclosion. Total numbers of GSCs analyzed are shown above each bar. The genotype of control flies shown in A and C is dinr339/+. (TIF) [file pgen.1004888.s014.tif]

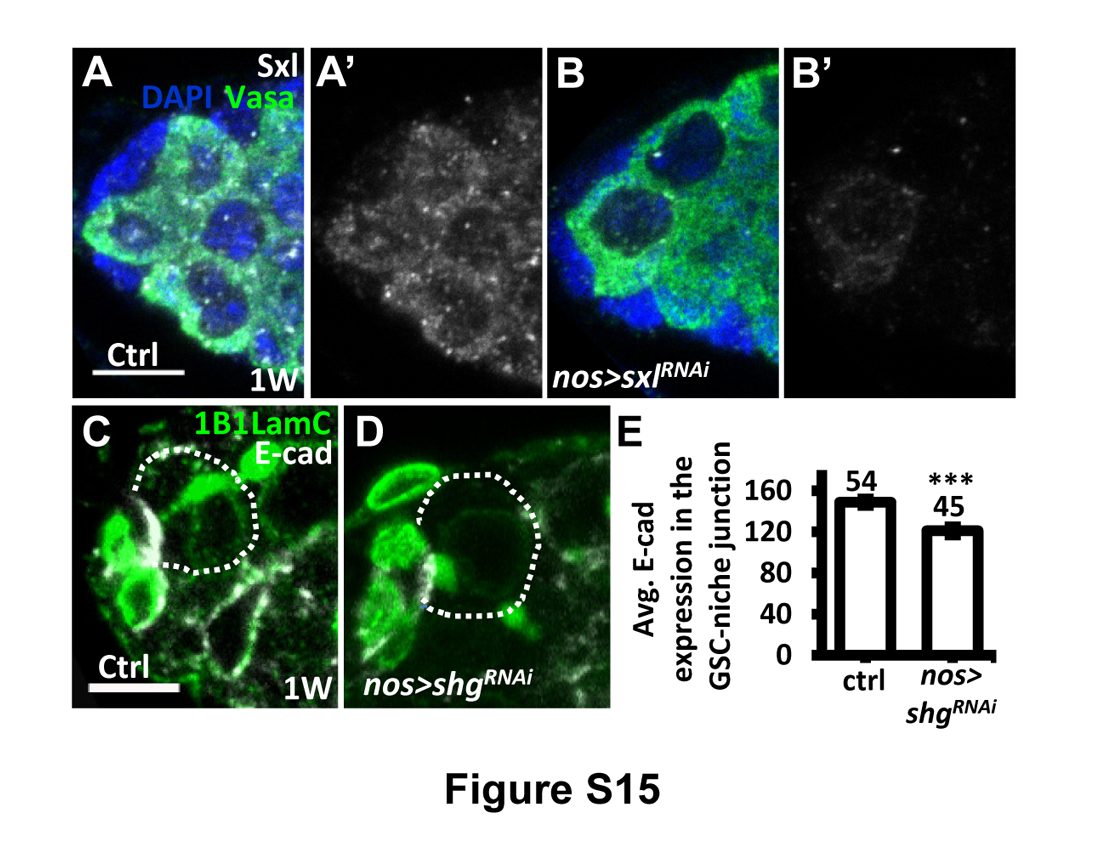

Supplement: S15 Fig — The sxlRNAi and shgRNAi constructs efficiently disrupt Sxl and E-cadherin in the germline. (A and B) One-week (w)-old ctrl (A) and sxl-knock down germaria (B) with Vasa (green, germ cells), Sxl (gray), and DAPI (blue, DNA) labels. A′ and B′ show the Sxl channel only. (C and D) Three-dimensional reconstructed images of one-week-old ctrl (C) and shg-knock down germaria (D) with LamC (green, nuclear envelopes of cap cells), 1B1 (green, fusomes), and E-cadherin (E-cad, gray) labels. Dashed lines indicate the position of GSCs. Scale bars: 5 µm. (E) Average (avg.) E-cad intensity at the junction between GSCs and their niche in 1-w-old ctrl and nos>shgRNAi germaria. Total GSCs analyzed are shown above each bar. ***, P<0.001; error bar, SEM. The genotype of the ctrl is tub-Gal80ts/+; nos-gal4-vp16/+ in A and tub-Gal80ts/UAS-mCD8-GFP; nos-gal4-vp16/+ in C and E. (TIF) [file pgen.1004888.s015.tif]
