# Supplementary material for: Notch Signaling Mediates the Age-Associated Decrease in Adhesion of Germline Stem Cells to the Niche
Source: PLoS Genet. 2014 Dec 18;10(12):e1004888. doi: 10.1371/journal.pgen.1004888 (PMC4270478; doi:10.1371/journal.pgen.1004888)
Supplement: S1 Table — Notch signaling controls GSC competition for niche occupancy via E-cadherin. (DOCX) [file pgen.1004888.s016.docx]

| **Table S1. Notch signaling controls GSC competition for niche occupancy via E-cadherin.** | | | | | | | | | | | | | | | | | | |  |
| --- | --- | --- | --- | --- | --- | --- | --- | --- | --- | --- | --- | --- | --- | --- | --- | --- | --- | --- | --- |
|  | |  |  |  |  |  |  |  | |  |  |  |  | |  |  |  | |  |
|  | |  |  |  |  |  |  |  | |  |  |  |  | |  |  |  | |  |
|  | | Clone | Total | % of germaria | |  |  |  | |  |  |  |  | |  | Total | Clonally-marked | | GSCs/ |
| Genotypes | | age | germaria | with GSC clone(s)**^a^** | | Fully GSC clone**^b^** | | | | | Partial GSC clone**^c^** | | | | | GSCs | GSCs (%)§ | | germaria |
| *FRT19A* Ctrl | | 1W | 639 | 28 ± 3 | (100)ǂ | 4 ± 3 | | (14)**^∏^** | | | 24 ± 3 | | | (86)**^∏^** | | 1310 | 16 ± 2 | (100)¥ | 2.0 ± 0.0 |
| 18℃ | | 2W | 636 | 27 ± 4 | (96 ± 8) | 4 ± 2 | | | (15) | | 23 ± 3 | | | (85) | | 1302 | 16 ± 3 | (100 ± 7) | 2.0 ± 0.1 |
|  | | 3W | 737 | 25 ± 4 | (89 ± 6) | 4 ± 1 | | | (16) | | 21 ± 3 | | | (84) | | 1465 | 15 ± 3 | (94 ± 8) | 2.0 ± 0.0 |
| *N^55e11^* | | 1W | 421 | 26 ± 2 | (100) | 6 ± 2 | | | (23) | | 20 ± 2 | | | (77) | | 770 | 16 ± 1 | (100) | 1.8 ± 0.1† |
| 18℃ | | 2W | 372 | 26 ± 1 | (100 ± 6) | 13 ± 1‖ | | | (50) | | 13 ± 2* | | | (50) | | 639 | 20 ± 1‖ | (125 ± 8*) | 1.7 ± 0.0† |
|  | | 3W | 392 | 30 ± 1 | (115 ± 7)¶ | 21 ± 3‖ | | | (70) | | 8 ± 4‖ | | | (30) | | 636 | 23 ± 0† | (144 ± 9‖) | 1.6 ± 0.1‖ |
| *N^54l9^* | | 1W | 427 | 21 ± 2 | (100) | 3 ± 1 | | | (14) | | 18 ± 1 | | | (86) | | 783 | 12 ± 2 | (100) | 1.8 ± 0.1* |
| 18℃ | | 2W | 332 | 26 ± 3 | (124 ± 4*)¶ | 10 ± 2‖ | | | (38) | | 16 ± 3 | | | (62) | | 592 | 18 ± 3* | (140 ± 8‖) | 1.8 ± 0.1‖ |
|  | | 3W | 351 | 24 ± 4 | (116 ± 10)¶ | 15 ± 2† | | | (63) | | 9 ± 3‖ | | | (37) | | 594 | 19 ± 1‖ | (158 ± 6†) | 1.7 ± 0.0‖ |
| *FRT19A* Ctrl | | 1W | 489 | 18 ± 4 | (100) | 3 ± 2 | | | (17) | | 15 ± 5 | | | (83) | | 1014 | 10 ± 2 | (100) | 2.1 ± 0.1 |
|  | | 2W | 509 | 20 ± 5 | (111± 5) | 4 ± 1 | | | (20) | | 16 ± 5 | | | (80) | | 956 | 12± 3 | (120 ± 8*) | 1.9 ± 0.1 |
|  | | 3W | 351 | 16 ± 2 | (89 ± 5) | 5 ± 2 | | | (31) | | 12 ± 3 | | | (69) | | 611 | 11 ± 0 | (110 ± 11) | 1.8 ± 0.2 |
| *N^AXE2^* | | 1W | 715 | 20 ± 8 | (100) | 4 ± 2 | | | (20) | | 17 ± 6 | | | (80 | | 1340 | 12 ± 5 | (100) | 1.9 ± 0.0† |
|  | | 2W | 698 | 14 ± 4 | (70 ± 8*) | 5 ± 1 | | | (36) | | 10 ± 4 | | | (64) | | 1140 | 10 ± 2 | (83 ± 11) | 1.6 ± 0.1 |
|  | | 3W | 415 | 12 ± 4 | (60 ± 8‖) | 8 ± 2 | | | (67)**ǂ** | | 4 ± 2 | | | (33) | | 595 | 12± 3 | (75 ± 6*) | 1.4 ± 0.1† |
| *sxl^fs3^* | | 1W | 150 | 21 ± 3 | (100) | 8 ± 4 | | | (44) | | 10 ± 4 | | | (56) | | 244 | 15 ± 4 | (100) | 1.6 ± 0.1† |
|  | | 2W | 200 | 12 ± 9 | (55 ± 36) | 9 ± 11 | | | (50) | | 9 ± 11 | | | (50) | | 273 | 13 ±10 | (78) | 1.4 ± 0.1† |
|  | | 3W | 235 | 7 ± 5 | (31 ± 20†) | 6 ± 5 | | | (85)ǂ | | 1 ± 1 | | | (15)ǂ | | 350 | 6 ± 4* | (36*) | 1.5 † 0.0† |
| *FRT80B* Ctrl | | 1W | 807 | 19 ± 3 | (100) | 2 ± 2 | | | (11) | | 17 ± 3 | | | (89) | | 1828 | 11 ± 0 | (100) | 2.2 ± 0.1 |
|  | | 2W | 741 | 14 ± 3* | (74 ± 8†) | 3 ± 1 | | | (21) | | 10 ± 2‖ | | | (79) | | 1519 | 12 ± 5 | (73 ± 2†) | 2.1 ± 0.1 |
|  | | 3W | 654 | 13 ± 0‖ | (68 ± 8‖) | 5 ± 2 | | | (38) | | 9 ± 2‖ | | | (62) | | 1330 | 9 ± 1 | (82 ±12) | 2.0 ± 0.2 |
| *fng^13^* | | 1W | 1095 | 18 ± 3 | (100) | 4 ± 2 | | | (22) | | 14 ± 4 | | | (78) | | 2263 | 10 ± 1 | (100) | 2.0 ± 0.1 |
|  | | 2W | 1058 | 17 ± 4 | (94 ± 7 ) | 9 ± 3‖ | | | (53) | | 8 ± 2* | | | (46) | | 1825 | 13 ± 3 | (130 ± 7*) | 2.0 ± 0.2 |
|  | | 3W | 782 | 12 ± 4 | (67 ± 9‖) | 7 ± 4 | | | (58) | | 5 ± 3‖ | | | (42) | | 1292 | 10 ± 4 | (100 ± 16) | 1.8 ± 0.2 |
| *fng^L73^* | | 1W | 1070 | 20 ± 4 | (100) | 6 ± 1 | | | (30) | | 14 ± 3 | | | (70) | | 2006 | 13 ± 3 | (100) | 1.9 ± 0.1‖ |
|  | | 2W | 904 | 14 ± 3* | (70 ± 9) | 8 ± 1‖ | | | (57) | | 6 ± 2‖ | | | (43) | | 1599 | 11 ± 1 | (85 ± 12) | 1.9 ± 0.1* |
|  | | 3W | 630 | 18 ± 5 | (90 ± 5) | 14 ± 2† | | | (78) | | 5 ± 2‖ | | | (22) | | 1025 | 16 ± 3 | (123 ±10*) | 1.8 ± 0.1 |
| *FRT82B* Ctrl | | 1W | 477 | 37 ± 5 | (100) | 7 ± 1 | | | (19) | | 30 ± 6 | | | (81) | | 1003 | 22 ± 3 | (100) | 2.1 ± 0.1 |
|  | | 2W | 351 | 34 ± 3 | (92 ± 5 | 9 ± 2 | | | (26) | | 25 ± 5 | | | (74) | | 740 | 20 ± 2 | (91 ± 8) | 2.2 ± 0.2 |
|  | | 3W | 482 | 32 ± 6 | (86 ± 5) | 12 ± 7 | | | (38) | | 20 ± 4 | | | (62) | | 972 | 21 ± 5 | (95 ± 10) | 2.0 ± 0.0 |
| *dinr^339^* | | 1W | 337 | 36 ± 3 | (100) | 5 ± 1 | | | (14) | | 31 ± 3 | | | (86) | | 712 | 19 ± 2 | (100) | 2.1 ± 0.1 |
|  | | 2W | 295 | 27 ± 4* | (75 ± 4‖) | 7 ± 6 | | | (26) | | 20 ± 9 | | | (74) | | 601 | 17 ± 3 | (89 ± 9) | 2.0 ± 0.2 |
|  | | 3W | 336 | 14 ± 2† | (39 ± 4†) | 6± 2 | | | (43)**ǂ** | | 8 ± 2† | | | (57) | | 644 | 12 ± 2‖ | (63 ± 8†) | 1.9 ± 0.1 |
| *FRT19A* Ctrl | | 1W | 348 | 23 ± 4 | (100) | 6 ± 4 | | | (22) | | 17 ± 3 | | | (78) | | 712 | 14 ± 3 | (100) | 2.0 ± 0.1 |
|  | | 2W | 395 | 21 ± 3 | (93 ± 3) | 7 ± 3 | | | (27) | | 14 ± 1* | | | (77) | | 675 | 13 ± 3 | (99 ±) | 1.9 ± 0.1 |
| *N^55e11^* | | 1W | 430 | 35 ± 10 | (100) | 20 ± 8 | | | (61) | | 15 ± 2 | | | (39) | | 810 | 24 ± 8 | (100) | 1.9 ± 0.2* |
|  | | 2W | 276 | 45 ± 12 | (130 ± 10)¶ | 36 ± 10 | | | (88) | | 9 ± 4* | | | (12) | | 451 | 41 ± 12* | (172 ±14†) | 1.6 ± 0.2* |
| *N^55e11^;shg^RNAi^* | | 1W | 207 | 41 ± 11 | (100) | 19 ± 2 | | | (46) | | 22 ± 11 | | | (54) | | 390 | 27 ± 3 | (100) | 1.9 ± 0.1* |
|  | | 2W | 179 | 37 ± 3 | (95 ± 7) | 23 ± 4 | | | (59) | | 15 ± 3 | | | (41) | | 270 | 30 ± 3 | (113 ± 8) | 1.5 ± 0.1* |

Flies were cultured on a standard food with wet yeast paste at 25℃, except *Notch (N)* loss–of-function mutant mosaic flies and their controls, which were cultured at 18℃ to avoid lethality. Food was changed daily until dissection. Notably, 3 weeks after clone induction, half of the *N^55e11^* and *N^5419^* mutants housed only one mutant GSC each; this was probably due to the GSC niches of these mutants carrying only one copy of functional *N*, and thereby affecting GSC maintenance. This is supported by the observation that the average GSC number per germarium was decreased. Similarly, *fng* and *dinr* mutant germaria also carried fewer GSCs as compared to control, a consequence of their role in Notch activation in the niche for its maintenance (Hsu and Drummond-Barbosa, 2011; Yang et al., 2013). The reduced number of GSCs was also observed for *N* gain-of-function (*N^AXE2^*) mutant GSCs, suggesting that excess Notch signaling in GSCs forces their removal from the niche.

**a**, Germaria carrying at least one clonally-marked GSC; **b**, germaria carrying GSCs that are all clonally-marked; **c**, germaria carrying GSCs that are not all clonally-marked.

**ǂ** Percentage of initial (1W) germaria carrying GSC clone(s) remaining at 2W and 3W; **∏** Proportion of germaria carrying fully (b) or partial clone (c) GSC clones; **¥** Percentage of total GSCs that are clonally-marked relative to that at 1W.

¶ The observed increase (relative to the normal clone induction rate) in the proportion of germaria carrying *N* mutant GSCs at 2 or 3 weeks after clone induction (ACI) resulted from the exclusion of degenerated *N* mutant germaria (in which germ cells were depleted); this was probably a consequence of partial disruption of the *N* mutant heterozygous niche, as reflected by the decreased number of GSCs.

**ǂ** The increased proportion of *N^AXE2^* and *dinr^339^* mutant mosaic germaria carrying fully GSC clones was mainly due to the decreased population of germaria carrying GSC clones (small denominator), which resulted from the loss of *N^AXE2^* and *dinr^339^* mutant GSCs.

***** Significant difference relative to initial value (1W): *P*<0.05; ‖ Significant difference relative to initial value (1W): *P*<0.01; † Significant difference relative to initial value (1W): *P*<0.001. Significant differences shown in the column “average GSCs per germaria” are relative to the controls.
